# Characterization of disomic alien addition lines from an intersubgeneric cross between *Glycine max* and *G. tomentella* and phenotypic variation within those lines and their progenies

**DOI:** 10.1007/s11103-026-01727-5

**Published:** 2026-08-28

**Authors:** Sufei Wang, Ram Singh, Gopal Battu, Mohammad Belaffif, Steven J. Clough, Matthew Hudson, Randall Nelson

**Affiliations:** 1 Department of Crop Sciences, University of Illinois Urbana-Champaign, 1101 W. Peabody Dr., Urbana, IL USA; 2 DOE Center for Advanced Bioenergy and Bioproducts Innovation, 1206 W. Gregory Drive, Urbana, IL, USA; 3 Present Address: Bayer U. S. - Crop Science, Seminis Vegetable Seeds, 500 Lucy Brown Lane, San Juan Bautista, CA USA; 4 Present Address: HudsonAlpha Institute for Biotechnology, 601 Genome Way Northwest, Huntsville, AL USA; 5 Soybean/Maize Germplasm, Pathology, and Genetics Research Unit, US Department of Agriculture–Agricultural Research Service, Urbana, IL USA

**Keywords:** Disomic alien addition lines, *Glycine max*, *Glycine tomentella*, Genetic diversity, Intersubgeneric hybrid, Genomic in situ hybridization, Fluorescent in situ hybridization

## Abstract

Disomic alien addition lines (DAALs, 2n = 42) were obtained from an intersubgeneric cross between *Glycine max* [L.] Merr. cv*.* Dwight (2n = 40, G1G1) and *Glycine tomentella* Hayata (PI 441001, 2n = 78, D3D3CC). These DAALs had two homologous *G. tomentella* chromosomes. In some progenies of the DAALs the extra *G. tomentella* chromosomes were eliminated. One objective of this research was to identify differences in gene expression between a DAAL (LG13-7552) and one of its 2n = 40 progenies (LG12-7063). RNA-sequencing documented that many genes critical to fundamental plant growth and related to stress and defense responses were differentially expressed. DNA and RNA-sequencing data showed that the gray pubescence of LG12-7063 was due to a novel adenine deletion in the *T* locus. When the DAALs were grown in the field, progeny were identified that possessed characteristics that were absent in both parents. Additional objectives of this research were to document this phenotypic variation and to determine the cause of these changes. All off-type progenies lost their *G. tomentella* chromosomes and reverted to the normal soybean chromosomal state (2n = 40). Significant variation was observed among the 2n = 40 progenies for the several qualitative and quantitative traits. Field research documented an average reversion frequency within the DAAL rows of 4%. Genotypic data showed that the revertants (2n = 40) differed from the sister DAAL lines by mostly heterozygous polymorphisms throughout the genome.Natural cross-pollination was the most probable cause of observed variation but the reasons for the elevated level of cross pollination and the mechanisms for long distance pollen transfer remain unknown.

**Supplementary Information:**

The online version contains supplementary material available at https://doi.org/10.1007/s11103-026-01727-5.

## Key Message

Alien addition lines from crossing *Glycine max* and *Glycine tomentella* differentially expressed genes related to stress and defense responses. Some progenies lost the alien chromosomes. Cross pollination caused this change but the mechanisms for elevated cross pollination are unknown.

**Supplementary Information:**

The online version contains supplementary material available at https://doi.org/10.1007/s11103-026-01727-5.

## Introduction

The genus *Glycine Willd.* has two subgenera; the subgenus *Soja (Moench) F.J. Herm.* (annuals) and the subgenus *Glycine* Willd. (perennials). The subgenus *Soja* contains two annual species, soybean (*Glycine max* [L.] Merr.), a critically important worldwide food, bioproduct and biofuel crop, and wild soybean (*G. soja* Seib. & Zucc.), and each contains 2n = 40 chromosomes and are included in the primary gene pool. The subgenus *Glycine* contains 26 perennial species that are considered the tertiary gene pool of soybean (Harlan and de Wet 1971).

Among the few perennial species that have been successfully hybridized with *G. max* (Grant et al. 1986; Newell et al. 1987), *Glycine tomentella* Hayata is unique, because it is a polyploid complex that has four cytotypes including aneudiploid (2n = 38), diploid (2n = 40), aneutetraploid (2n = 78) and tetraploid (2n = 80) (Doyle and Brown 1985). Perhaps due to its complexity and variability in genetic composition, growth habits, and geographical distribution, *G. tomentella* harbors numerous desirable traits, including disease resistance (Burdon and Marshall 1981; Zheng et al. 2005), pest resistance (Zhuang et al. 1996; Bauer et al. 2007), and stress resistance (White et al. 1990; Kao et al. 2006), which makes it a promising source for soybean improvement. The earliest wide hybridizations between soybean and *G. tomentella* were in 1979 (Broue et al. 1979; Ladizinsky et al. 1979). Additional research developed backcross-derived progenies (Singh et al. 1990) and produced fertile progeny from backcrosses between soybean and perennial *Glycine* (Singh and Nelson 2015)*.* The first confirmed disomic alien addition lines (DAALs) (2n = 40 *G. max* chromosomes + 2 *G. tomentella* chromosomes) from crosses between *G. max* Dwight and *G. tomentella* PI 441001 were identified in 2008 (Singh and Nelson 2015). Since these DAALs (2n = 42) were derived from a single monosomic alien addition line (MAAL), each plant carried two homologous chromosomes from *G. tomentella* and 20 pairs of soybean chromosomes.

The DAALs were first grown in the field in 2008 (Singh and Nelson 2015) and from 2008 through 2016, single plants were harvested each year from progenies of these original rows. The DAALs were much shorter and had delayed maturity compared to Dwight. A 2n = 40 progeny, LG12-7063, was selected to compare its transcriptome profile to that of the progenitor DAAL (LG13-7552) via RNA-sequencing. Both LG12-7063 and LG13-7552 were derived from the same F3 row (F3 Dwight (4) × PI 441001) and were bulk harvested after 3 and 4 generations, respectively, of annually harvesting single plants. Since our objective was to determine the effect of the 2 *G. tomentella* chromosomes, LG12-7063 was selected for comparison to the DAAL to reduce the effects of any *G. tomentella* introgression into the soybean genome. Genomic in situ hybridization with *G. tomentella* DNA was used to demonstrate that LG12-7063 had no *G. tomentella* chromosomes.

Many other DAAL-derived progenies were selected as morphologically distinct from the progenitor DAAL and both the recurrent soybean parent Dwight and the perennial parent *G. tomentella* PI 441001. The range of phenotypic variation among the selfed progenies of the DAALs was much greater than had been observed in other populations derived from wide hybridizations between *G. max* and *G. tomentella*. The objectives of this research were to identify differences in gene expression between a DAAL (LG13-7552) and one of the 2n = 40 DAAL progeny (LG12-7063), to document the phenotypic variation among the DAAL-derived soybean lines and determine the genetic cause of the variation.

## Materials and methods

### Field research and DNA and RNA sequencing

All field research was conducted at the University of Illinois Crop Science Research and Education Center at Urbana-Champaign. All the DNA and RNA sequencing including enrichment for mRNA were performed at the Roy J. Carver Biotechnology Center at the University of Illinois at Urbana-Champaign.

### Phenotypic variation among DAALs and derived progenies

The DAALs were derived from a confirmed 2n = 41 plant (F2 Dwight (3) × PI 441001) grown in the greenhouse in 2007 (Singh and Nelson 2015). In selfing 2n = 41 plants, the chromosome number of the *G. max* genome would not change and the three segregation options in the progeny would be zero, 1, or 2 *G. tomentella* chromosomes. Chromosome counts confirmed the lines with 2n = 42 chromosomes (two homologous chromosomes from *G. tomentella* and 20 pairs of soybean chromosomes) (Singh and Nelson 2015).

In 2011, single plants with a different phenotype from the DAAL line were harvested. In 2012, approximately 1400 single plant progenies (F6 Dwight (4) × PI 441001) that trace back to three original DAALs were planted. Phenotypically uniform rows that were morphologically different from a typical DAAL were bulk harvested. Field trials of 73 lines were conducted in a randomized complete block design with two replications in 2013 and four replications in 2014. The rows were 1.2 m long and 75 cm between rows with 36 viable seeds planted per row. Two DAALs (LG12-11684 and LG12-7663) and Dwight were included in each replication as checks. Seeds from each row were bulk harvested to record seed traits. LG13-7552 was not available for the 2013 test but was included in 2014. Because it was an unbalanced design, we calculated LSMEANS for each trait for each entry as well as the least significant differences at α = 0.05 for each trait for pair-wise comparison between entries.

Phenotypic data included qualitative traits: flower, pubescence, pod, seed coat, and hilum color, and stem termination; and quantitative traits: plant height (cm), lodging score (from 1 to 10, with 1 being erect), maturity date (days after May 31), 100 seed weight (g), and seed protein and oil concentrations (g/kg). Protein and oil concentrations were measured using a Perten Diode Array 7200 near-infrared spectroscopy instrument (NIR, Perten Instrument, Sweden) on all seed lots with a yellow seed coat and adjusted to 13% moisture. Statistical models and analyses for protein and oil concentration, plant height, lodging score and maturity included entries as a fixed effect, and replications and years as random effects.

### Frequency of qualitative trait changes

Field experiments to document the frequency of phenotypic changes within DAALs were conducted from 2013 through 2016. Of specific interest were tawny pubescence plants from gray pubescence lines, indeterminate plants from determinate lines, and yellow seed coat plants from black seed coat lines.

The lines that were monitored for changes in pubescence color were derived from a confirmed 2n = 41 plant (F2 Dwight (3) × PI 441001) grown in the greenhouse in 2007 (Singh and Nelson 2015). The 2008 plant row had a distinctly different morphology from either parent and was later in maturity and shorter in height than Dwight and had rugose leaflets. Twelve single plants were harvested to determine the genetic stability of the line. In 2009, plants that more closely resembled the soybean parent were observed within some progeny rows. One of these plants produced a progeny row in 2010 that was segregating for height, maturity, and pubescence color. Fourteen single plants were harvested from that row and were grown as progeny rows in 2011. Single plants were harvested from 13 of those rows. In 2012, some of the rows appeared uniform. They were bulk harvested to create 93 lines that were planted in a 2-replication test in 2013 to assess the range of diversity in this population. In 2013, variation was observed within some of the previously uniform lines and single plants were harvested again from these rows. From 2014 to 2016, these single plants were used to initiate the documentation of the within line changes from gray to tawny pubescence color.

The lines that produced data for segregation for stem termination were derived from 2n = 42 progenies grown in the field in 2008 from a 2n = 41 greenhouse-grown plant the previous year. In 2009 and 2010 all plants were indeterminate but single plants were harvested because of phenotypic variation for height and maturity. In 2011 determinate plants were observed within one row. From a single determinate plant harvested in 2012, segregation for determinacy was observed in 2013. Single plants were harvested in 2014 and the determinate plants were used to initiate the documentation for determinacy changes in 2015 and 2016.

The lines that were used to monitor changes in seed coat color were derived from the same derived 2n = 42 plant as those used to document change in stem termination. Single plants were harvested from 2009 through 2011. In 2012, plants with black seed coats were identified. Single plant progenies were grown in 2013 and observations on plant reverting from black to yellow seed coat were recorded from 2014 to 2016.

### Frequency of revertants in 2n = 42 lines

Each year, the number of deviant plants and the number of typical DAAL plants were counted in rows that were derived from single DAAL plants the previous year. In rows derived from some deviant plants, segregation ratios were calculated for seed coat color, pubescence color and stem termination. Seeds for this study came from single plants harvested from DAAL rows in which phenotypic change occurred the previous year. Seeds from each plant were carefully threshed to avoid contamination with seeds from other plants. The number of plants with each phenotype for each trait of interest in each plant row was recorded, and the deviation rate was calculated as the ratio between the number of deviants that had phenotypic changes from the progenitor and the total number of plants. Chi square tests were used to determine if the segregation among the progenies fit into an expected 3:1 Mendelian ratio.

### Cytogenetics

Root tips for chromosome counts from selected lines were collected from plants grown in a sand bench. Root tips were pre-treated with an 8-hydroxyquinoline solution (0.5 g/L) to cause chromosome contraction followed by a fixative containing 3 parts of ethanol and 1 part of glacial acetic acid and a hydrolyzing treatment of 1N HCl. After storing at room temperature for at least 24 h, root tips were twice washed with distilled H_2_O, hydrolyzed in 1N HCl at 60 °C and then washed again with distilled H_2_O for 10 min. Treated root tips were stained with Feulgen stain for one hour to create purple colored root tips and then stored in cold water. Before counting, the purple colored root tips were further stained with Carbol Fuchsin on a clear glass slide for observation under a compound microscope (Singh 2018).

### In situ hybridization

*G. tomentella* (PI 441001) genomic DNA was extracted using a modified CTAB protocol (Doyle and Doyle 1987; Porebski et al. 1997). *G. max* and *G. tomentella* seedlings were grown and the roots prepared (Gill et al. 2009) using pressurized nitrous oxide (Kato et al. 1999) to arrest the cells in Metaphase.

The genomic in situ hybridization (GISH) probe was generated by modifying the BAC FISH probe method (nick translation) (Findley et al. 2010) for use with genomic DNA. The probe was purified by passing it through a Centri-spin 50 column (Fisher NC9837373) as per the manufacturer’s instructions. 4ul (400 ng) of labeled genomic DNA was used per slide for GISH with LG13-7552 (2n = 42) and LG12-7063 (2n = 40). *G. max* centromeres were labeled using four Texas red-615 labelled probes (CentGm-2-G, CentGm-1-E, CentGm-1-AF, CentGm-2-M) (Integrated DNA Technologies, Inc) to soybean centromeric repeats CentGm1 and CentGm2 (Findley et al. 2010). 2 ng of each probe was combined into a 10ul hybridization mix for use in fluorescent in situ hybridization (FISH). Simultaneous FISH using Texas red labeled *G. max* centromeric probes and GISH using Fluorescein labeled *G. tomentella* genomic DNA were performed (Findley et al. 2010).

After hybridization, the slides were mounted in a 1:3 diluted Vectashield with DAPI (Vector Labs H1200) (diluted with Vectashield without DAPI (Vector Labs H1000)). The slides were viewed on a Leica DM2500 microscope. Three images from each of 3 channels from a single field of view (DAPI, Texas red and Fluorescein) were captured using a Canon Rebel XS camera. The 3-channel merged image was generated, cropped, and modified in Adobe Photoshop (CS5). Using the "Channels" toolbar, the red channel from the Texas red image was copied and pasted into the red channel of the DAPI image. Similarly, the green channel from the Fluorescein image was copied and pasted into the green channel of the DAPI image. The image was then cropped to include only one nuclear spread. Each channel was separately edited to remove cytosolic background signals. The image was then saved as a merged image. The red, blue, and green channels were then separated and saved as three different images, one for each channel.

### RNA sequencing and differential expression analysis

Seeds from LG13-7552 (2n = 42) and LG12-7063 (2n = 40) were grown in sand in a controlled environment (24 °C) independent from *G. tomentella* plants. Root tips were sampled for chromosome counts at the first unifoliolate leaf stage and then transplanted into individual pots. When second trifoliolate leaves of the transplanted plants were fully unfolded, all tissue above the first trifoliolate leaf of each plant was collected for RNA-Seq. Each line included three biological replicates. Tissue was flash-frozen in liquid nitrogen and total RNA was isolated using TRIzol Reagent and Phase Lock Gel-Heavy (Zou et al. 2005). The RNA was treated with DNase I (Ambion) and purified with the RNeasy kit (QIAGEN). Purified RNA samples were quality-checked using an Agilent®2100 Bioanalyzer™ (Agilent®, Santa Clara, CA, USA) and high-quality samples (> 1ug RNA) were sequenced using Illumina Hi-Seq 2500, generating 100-bp paired-end reads.

For reference-guided analysis, Novoalign maps each read to a specific location in the soybean genome and quantifies expression of known genes. Transcripts with known and theoretical splice junctions and a hard-masked soybean genome were generated from the *Glycine max* Williams 82 (Wm82.a2.v1, Schmutz et al. 2010) reference genome and indexed using USeq. Alignments from Novoalign (Novocraft Technologies, Selangor, Malaysia) were converted to genome coordinates using USeq SamTranscriptomeParser, and generated gene-level read counts using HTSeq (Anders et al. 2014). Novoalign aligned 331,328,696 paired-end reads to the Williams 82 reference genome, representing approximately 84% of the total reads generated. Of these aligned reads, approximately 72 million pairs with a mapping quality score (MapQ) below 10 were discarded due to low confidence. Among the low-quality alignments, 94% had a MapQ score of 3, indicating multi-mapping reads, likely reflecting the highly repetitive nature of the soybean genome. The Williams 82 reference genome contains 73,320 predicted gene models; excluding alternative splice isoforms, 54,175 were considered primary transcripts (Schmutz et al. 2010) and were used for downstream gene-level expression analyses.

Differential expression analysis was performed using DESeq (Anders and Huber 2010), with significance determined at an adjusted *p* value and false discovery rate (FDR) of 0.05 using the Benjamini–Hochberg correction. Principal component analysis was conducted within DESeq using all six samples. Differentially expressed genes (DEGs) were subjected to Gene Ontology (GO) singular enrichment analysis using agriGO (Du et al. 2010b), with the Williams 82 genome as the reference and GO annotations from 29,641 genes. GlymaID identifiers of DEGs were used to identify enriched GO categories.

Trinity de novo assembler allows for the study of the transcriptome without a reference genome and the detection of all genes, not limited to soybean, Assembled contigs from Trinity were annotated using a series of sequential BLAST searches (Altschul et al. 1998). All assembled contigs were first aligned by BLAST against the soybean primary transcript (*Glycine max* Wm82.a2.v1) to assign GlymaID identifiers based on top hit, and those mapping to previously described or predicted coding regions were excluded. The remaining contigs that did not align to any part of the soybean genome were considered non-soybean sequences and were aligned by tblastx to the NCBI nt sequence database (http://www.ncbi.nlm.nih.gov) to obtain an annotation, followed by blastx (E-value < 1e−06) against NCBI nr database for putative functional analysis.

Functional annotation of differentially expressed genes was performed by BLASTx against the NCBI nr protein database using predicted gene models in soybean reference genome. Top 10 hits for each GlymaID were obtained and the best hit with a descriptive annotation was assigned to each DEG to resolve ambiguous annotation and infer gene function.

In parallel, all contigs were blasted against the eudicots repetitive element database in Repbase (Jurka et al. 2005) using RepeatMasker (RM) (Smit and Hubley 2010). Contigs within the categories of “simple repeat” and “low complexity” were removed while the remaining contigs are considered as candidate transposable elements (TEs).

### *G. tomentella* transcriptome

*G. tomentella* root, cotyledon, unifoliate and trifoliate leaves, shoot tip and young flowers were flash frozen in liquid nitrogen. Total RNA was extracted using cetyltrimethylammonium bromide (CTAB) (Chang and Puryear 1993). An acid phenol–chloroform step was added before the chloroform-isoamyl alcohol step. mRNA was isolated using Qiagen Oligotex mRNA Midi kit (70042). 33 ng of mRNA from each tissue was pooled (~ 200 ng total) and sent to the sequencing center. There it was processed and sequenced on Illumina HiSeq2000 to obtain 72,095,156 reads (100 bp paired end). The reads were adaptor and quality trimmed using in-house scripts. They were used to assemble a transcriptome using abyss (1.2.4) (Simpson et al. 2009). In house scripts were used to pool and collapse multiple k-mer contigs. The transcriptome was then filtered with NCBIs Foreign Contamination Screen (FCS) suite of tools (FCS-adaptor and FCS-GX) to remove adaptor/vector sequences and foreign organism sequences. The final transcriptome had 76,440 contigs with an N50 of 1530 bp.

### Genetic research on three single-loci traits

Genomic DNA samples were extracted from bulked, young-leaf tissue of 8–10 seedlings using the CTAB method. The *T* locus, which encodes a flavonoid 3′-hydroxylase gene (F3′H) and controls pubescence color in soybean, was sequenced to determine if any sequence variation existed between selected lines. Primers were designed to amplify the promoter (F3h-1F and F3h-1R) and each exon region (F3h(2-4)-F and F3h(2-4)-R) of F3′H gene based on the Glyma.06G202300 gene sequence from the Williams 82 reference genome, to which Trinity assembled contig (c23278_g1) was aligned. The PCR amplicons were sequenced by the Sanger method.

**Table:** 

Primer name	Sequences (5′–3′)	Tm (°C)
F3h1-F	TGGTTTGAAGGAATGAAAGATG	55
F3h1-R	GCTAGCATGGGGCTTCAG	55
F3h2-F	TGAATTGTATTTCAAAGGCTCA	55
F3h2-R	TGCATAGGATATTCAGTGGACA	55
F3h3-F	TGGCAGCATTACGTTACCTTT	55
F3h3-R	TTGGCAATCCTACTATAAACGAG	55
F3h4-F	ACACCATTTCTCAAGTACAAAGG	55
F3h4-R	GGGGGTGTAATAAAAGAATGTATG	55

For the *Dt1* locus, we sequenced two approximately 1 kb PCR-amplified genomic regions of the *Gmtfl1* gene (Tian et al. 2010) using primers provided by J. Ma, Purdue University. GmTfl1-frag2F and GmTfl1-frag2R contained the SNP position corresponding to the *GmTfl1-ta* allele and GmTfl1-frag3F and GmTfl1-frag3R had the three SNPs for *GmTfl1-ab, GmTfl1-tb, and GmTfl1-bb*. PCR programs for the two fragments were the same as the reactions for F3′H gene, except the annealing temperature for GmTfl1-frag2 primer pair was 58 °C and for GmTfl1-frag3 pair was 53 °C. All amplified fragments were Sanger sequenced. Sequence alignment and variant detection were performed in Sequencher® (version 5.4.6 DNA sequence analysis software) and Clustal Omega (Sievers et al. 2011).

The CHS duplicated inverted repeat within the *I* locus (Clough et al. 2004) was analyzed using PCR amplication of nine PCR amplicons (Clough et al. 2004; Tuteja et al. 2004) with a modified two-stage touchdown PCR program. PCR products were visualized in 1% agarose gels.

### Genotyping-by-sequencing

Approximately 2000 single plant rows derived from the DAALs harvested in 2008 were planted in the field in 2015. From these rows, 185 single plants with morphologies distinct from the DAAL progenitor or the soybean parent were selected to plant in 2016 to represent the phenotypic variation. From those plots, 124 lines were selected for genotyping via genotyping-by-sequencing (GBS). A two-enzyme restriction digest, HindIII and MseI, was employed to construct a GBS library of DNA fragments with adaptors on each end of the restriction fragment (Poland et al. 2012). After restriction digestion and ligation, the DNA samples were purified using Agencourt AMPure XP beads (Beckman Coulter Life Sciences, Indianapolis, IN, USA) at a concentration designed to recover amplicons that are greater than 100 bp in length. The amplification thermal cycler program annealed at 98 °C for 30 s, followed by 15 cycles of 10 s at 98 °C, 30 s at 68 °C and 30 s at 72 °C, and then a 5 min final extension at 75 °C. The libraries were sequenced on a HiSeq2500 (Illumina, San Diego, CA, USA) with 100 base pair single end.

Illumina raw sequence and SNP callings was performed in a pipeline (Wickland et al. 2017) that used the publicly available software Stacks (Catchen et al. 2013) for demultiplexing, BWA (Li and Durbin 2009) for alignment, SAMtools (Li et al. 2009) for likelihood calculation, BCFtools for SNP calling, and VCFtools to filter minor allele frequency (Danecek et al. 2011). The obtained usable SNPs had a minimum read depth of 2, minor allele frequency of 0.05, and maximum missing data of 0.5.

### Illumina infinium HD assay

The genotypes of 21 revertants collected from different DAAL rows from 2013 to 2016 and DAAL sister lines from each year were profiled using BARCSoySNP6K_v2 Illumina Infinium HD Assay (SoySNP6K) (Song et al. 2013). This assay contains 6000 SNPs selected from the SoySNP50K Illumina Infinium Beadchip, a high-density SNP assay of 52,041 SNPs from soybean euchromatic and heterochromatic genomic regions.

Genetic distances were obtained from TASSEL Distance Matrix (Bradbury et al. 2007) that defines genetic distance as 1—identity by state, which is the probability of drawing identical alleles from two individuals at random. Lines with fewer than 40% genotyped markers were dropped from the distance calculation. The physical locations of SNP markers and genotypes across chromosomes were visualized in PhenoGram (Wolfe et al. 2013).

## Results

### Differential expression to explain phenotypic differences between LG13-7552 and LG12-7063

**Fig. 1:**
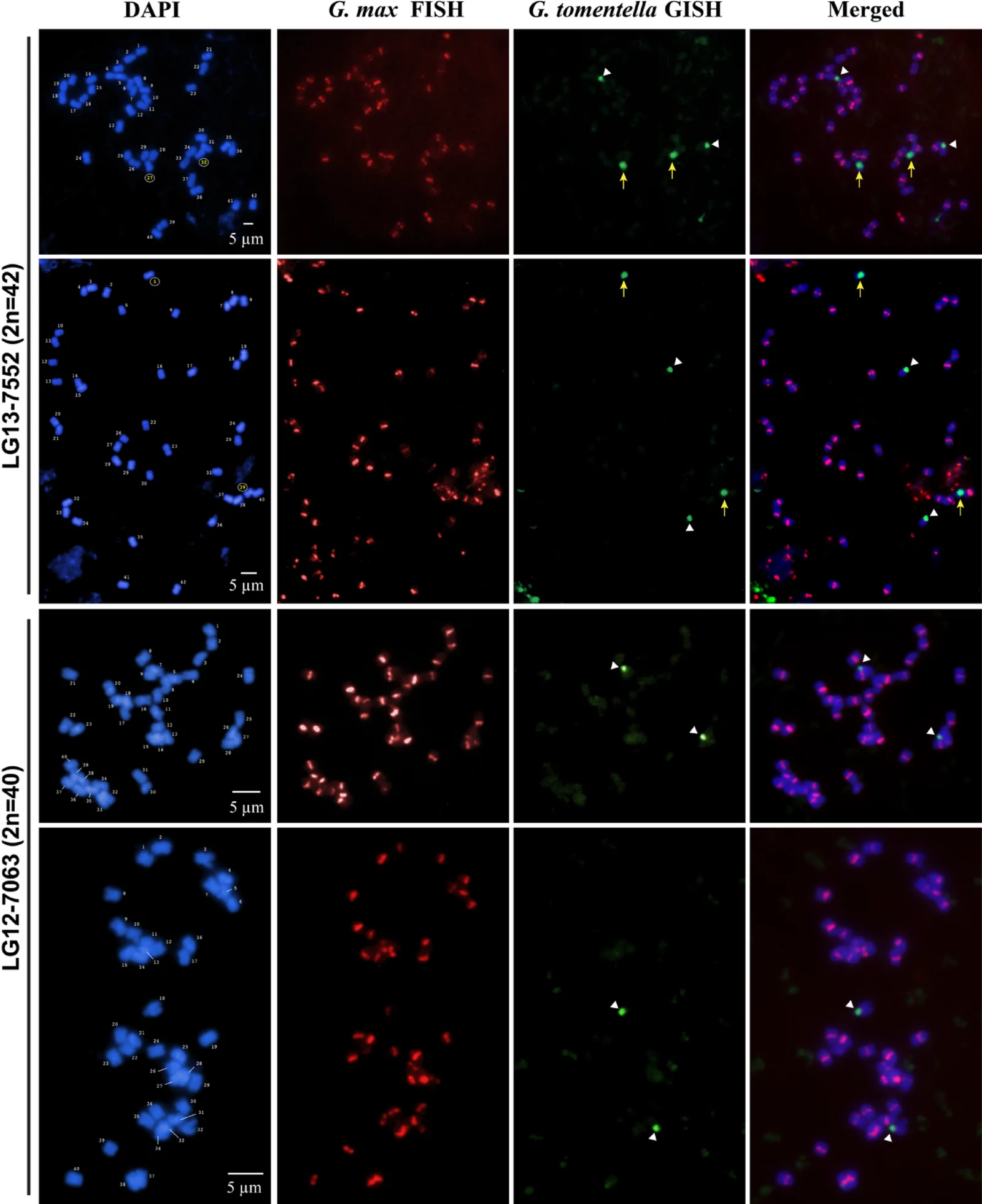
Fluorescent *in situ* hybridization (FISH) and genomic *in situ* hybridization (GISH) of LG13-7552 and LG12-7063. The top and bottom panels within each line show two different chromosome spreads. The blue DAPI stained chromosomes are the 1st column. The red FISH images using four Texas red labelled probes to *G. max* centromeric repeats are the 2nd column. The green GISH images using Fluorescein labelled *G. tomentella* genomic DNA are the 3rd column. The merged DAPI/FISH/GISH images are the 4th column. The yellow arrows indicate the *G. tomentella* derived chromosomes that stain with the GISH probe and not the *G. max* FISH probes. The white arrow heads point to the *G. max* Nucleolar Organizer Region (NOR) that cross-hybridize with the *G. tomentella* probes. The chromosomes are randomly numbered for counting only and are not genetically designated chromosome numbers. LG13-7552 has two *G.tomentella* chromosomes as evidenced by staining of 2 chromosomes with the GISH and 40 with the FISH probes and are circled in the numbering in the DAPI images (1st and 2nd row). LG12-7073 on the other hand has only 40 chromosomes, all of which show the *G. max* centromeric FISH staining (3rd and 4th row). Scale bars are 5 µm. DAPI is 4′,6-diamidino-2-phenylindole.

We field tested the two lines selected for RNA-Seq (LG13-7552, 2n = 42, and LG12-7063, 2n = 40) and two additional 2n = 42 lines to document the phenotypic differences between the 2n = 40 and the 2n = 42 lines (Table 1). The DAAL lines (2n = 42, LG12-11684, LG12-7663, LG13-7552) were very similar to each other but were very different from Dwight and LG12-7063 (2n = 40) (Table 1). The DAALs had tawny pubescence, yellow seed coats with black hila, brown pods, purple flowers and indeterminate stem termination. These qualitative traits were consistent with Dwight, the soybean parent, but the DAALs were much shorter compared to the soybean parent Dwight (56 to 60 vs 85 cm), and later in maturity (129 to 145 vs 114 days) (Table 1). LG12-11684 was not different in protein concentration from Dwight but LG12-7663 and LG13-7552 were significantly higher (Table 1). All DAALs were significantly lower in oil concentration than Dwight (Table 1). There was no variation for plant height or lodging among the DAALs (Table 1). Besides the listed traits, we also observed that the DAALs had shorter internodes, reduced pod set, rugose leaves with delayed leaf abscission, and green stem at maturity. We confirmed the presence of two *G. tomentella* chromosomes in LG13-7552 and their subsequent loss in LG12-7063 using FISH and GISH (detailed below and Fig. 1).

**Table 1:** Chromosome number and trait means for lines representing the range of diversity among entries derived from disomic alien addition lines and grown in 2013 (2 replications) and 2014 (4 replications) at Urbana, Illinois. Qualitative data for PI441001 were not collected in this research but are provided for comparison purposes.

Entry	2n^a^	Pub^b^	SC^c^	HC^d^	FC^e^	PC^f^	ST^g^	Pro^h^	Oil^h^	Hgt^i^	Ldg^j^	Mat^k^
PI441001	78	T	Bl	Bl	P	Br	–^l^	–	–	–	–	–
LG12-11684	42	T	Y	Bl	P	Br	Ind	409	201	60	1.9	131
LG12-7663	42	T	Y	Bl	P	Br	Ind	423	193	56	1.9	129
LG13-7552	42	T	Y	Bl	P	Br	Ind	416	192	58	1.9	130
Dwight	40	T	Y	Bl	P	Br	Ind	389	216	85	2.8	114
LG12-11711	40	T	Y	Bl	P	Tn	Ind	377	215	71	3.0	111
LG12-11954	40	T	Y	Br	P	Tn	Det	422	185	86	3.6	125
LG12-12024	40	T	Y	Br	P	Tn	Ind	394	213	83	2.9	110
LG12-12125	40	G	Y	Ib	P	Tn	Det	408	199	65	1.7	116
LG12-7063	40	G	Y	Bf	W	Br	Ind	486	168	99	6.3	130
LG12-7072	40	G	Y	Ib	P	Tn	Ind	445	184	93	5.1	126
LG12-7074	40	T	Y	Bl	P	Tn	Ind	407	192	86	5.6	122
LG12-7076	40	T	Y	Bl	P	Br	Ind	420	195	89	4.8	117
LG12-7080	40	T	Y	Bl	P	Tn	Ind	418	192	105	4.5	125
LG12-7081	40	T	Y	Bl	P	Tn	Ind	409	195	102	4.5	115
LG12-7120	40	T	Y	Bl	P	Tn	Ind	409	189	88	6.7	124
LG12-7127	40	T	Y	Bl	P	Br	Ind	412	191	103	6.6	124
LG12-7155	40	T	Y	Bl	P	Tn	Ind	431	183	93	7.2	118
LG12-7157	40	G	Y	Ib	P	Tn	Ind	426	184	100	6.7	125
LG12-7159	40	T	Y	Bl	P	Tn	Ind	420	190	99	6.9	118
LG12-7174	40	G	Y	Ib	P	Br	Ind	415	188	99	5.6	127
LG12-7177	40	G	Y	Bf	P	Br	Ind	402	215	110	3.1	126
LG12-7182	40	G	Y	Bf	P	Br	Ind	389	218	100	2.9	119
LG12-7191	40	G	Y	G	P	Tn	Ind	393	212	118	3.2	116
LG12-7196	40	G	Y	Bf	P	Br	Ind	404	208	120	3.2	125
LG12-7214	40	T	Y	G	P	Br	Ind	399	213	115	3.8	115
LG12-7227	40	G	Y	Ib	P	Br	Ind	408	200	117	3.0	128
LG12-7248	40	G	Y	Y	P	Tn	Ind	371	223	104	3.7	114
LG12-7296	40	T	Y	G	P	Tn	Ind	407	220	112	3.7	112
LG12-7298	40	T	Y	G	P	Br	Ind	396	214	102	4.1	109
LG12-7335	40	G	Y	Ib	P	Br	Ind	381	221	104	3.4	112
LSD_0.05_^m^								20	14	13	1.6	7

^a^Chromosome number

^b^Pubescence color; T: Tawny; G: Gray

^c^Seed coat color; Y: Yellow; Bl: Black

^d^Hilum color; Bf: Buff; Br: Brown; Bl: Black; G: Gray; Ib: Imperfect black; Y: Yellow

^e^Flower color; P: Purple; W: White

^f^Pod Color; Br: Brown; Tn: Tan

^g^Stem termination; Det: Determinate; Ind: Indeterminate

^h^Protein concentration and oil concentration (g/kg) on a dry weight basis

^i^Height in cm

^j^Lodging score, 1 being upright and 10 being prostrate

^k^Maturity, days after May 31st

^l^Data not collected

^m^LSD were calculated from all entries in the 2013 and 2014 field study

The 2n = 40 progeny selected for RNA-Seq, LG12-7063, was different from the progenitor DAAL and both parents Dwight and PI 441001 for most qualitative and quantitative traits (Table 1). It had gray pubescence, buff hilum color, and white flower color, which were traits absent in the progenitor DAAL and both parents. The protein concentration of LG12-7063 was higher than the highest DAAL and Dwight (486 g/kg vs 425 g/kg and 389 g/kg, respectively), while oil concentration was lower (168 g/kg vs 180 g/kg and 216 g/kg respectively). LG12-7063 was taller, lodged more and was later maturing than Dwight (Table 1).

Novoalign genome alignment and Trinity de novo assembly are used to yield a comprehensive view of the transcriptome by leveraging the strength of the two different approaches. Using Novoalign, the total number of captured, expressed genes in LG13-7552 and LG12-7063 from mapping to soybean genome Williams 82 was 41,602 regardless of the line, which is 77% of the total predicted. The statistical analysis identified 2499 of these transcripts as being differentially expressed genes (DEGs) between the DAAL (LG13-7552) and the 2n = 40 progeny line (LG12-7063). There were 1192 DEGs in higher abundance in LG12-7063 than in LG13-7552 and 1179 DEGs in lower abundance. Among the detected DEGs, 128 were uniquely transcribed in LG12-7063, whereas 79 DEGs were unique in LG13-7552. These uniquely expressed genes were excluded from the discussion of DEGs in alignment, because they resulted from mapping to the sequence of soybean reference genome, which is different from the sequence of the recurrent parent Dwight, and the zero read counts were more likely due to high mapping stringency and/or insufficient sequencing depth. Among the 2292 DEGs that were expressed in both lines, 641 had expression levels of at least a fourfold difference.

Principal component analysis using Novoalign mapped sequences from all six DAAL and 2n = 40 plants provided general information on the between- and within-group variances of RNA-sequencing reads from the six plants (Supplemental Fig. 1). Principal component 2 (PC2) is the vector that represents the within group variance, which may consist of the effects of several influential genes or a group of differentially expressed genes affected by extrinsic microenvironment factors. Although there is considerable variance within each chromosomal group, PC1 can clearly separate LG13-7552 and LG12-7063 on one dimension and represents the differential expressions of genes that are responsible for the main differences between the DAAL and the 2n = 40 progeny.

To analyze the RNA-Seq data in a manner that would allow for the study of all the sequences independent from a reference genome and detection of non-soybean genes, we used the software Trinity. It builds full-length transcript sequences from overlapping RNA-Seq data and uses the assembled contigs as reference. Trinity identified 133,872 contigs from LG13-7552 and LG12-7063, and 4,206 contigs (considered as genes) were differentially expressed between the two lines. Among the total DEGs, 2894 had expression in both lines, while the expression level of 1457 contigs was higher in LG12-7063 than in LG13-7552 and 1437 contigs lower in LG12-7063. There were 989 contigs expressed only in LG13-7552, which is much higher than the number of genes expressed only in LG12-7063 (323) (Table 2).

**Table 2:** Summary of functional annotations of contigs from Trinity de novo assembly using merged RNA sequencing data of the DAAL (LG13-7552) and the 2n = 40 progeny (LG12-7063).

	Total Contigs^a^	Hits to Primary Transcript^b^	Hits to Non-primary Transcript^c^	*G. tomentella*^d^	Repetitive Element Database^e^
Total	133,872	61,569	69,525	213	26,136
DEGs^f^	4206	1563	–^g^	51	1077
Up-regulated in LG13-7552 (DAAL)	1437	701	–	0	399
Up-regulated in LG12-7063 (2n = 40)	1457	862	–	0	242
Only in LG13-7552 (DAAL)	989	–	–	51	342
Only in LG12-7063 (2n = 40)	323	–	–	0	94

^a^Total number of de novo contigs from Trinity de novo assembly

^b^Number of de novo contigs that hit to the primary transcript of the soybean reference genome Williams 82 from BLAST

^c^Number of de novo contigs that hit to the soybean reference genome Williams 82 other than the primary transcript from BLAST

^d^Number of de novo contigs that were not identified in the soybean reference genome Williams 82 but hit *G. tomentella* sequences publicly deposited in National Center for Biotechnology Information (NCBI) database from BLAST

^e^Number of de novo contigs that were identified to be repetitive elements by RepeatMasker (RM)

^f^Differentially expressed genes

^g^Dash (–) indicates contig number does not apply to the category

In the BLAST results, 61,569 Trinity contigs had hits against the primary transcript of the soybean reference genome, which made up 46% of the total contigs. Of these, 1563 contigs with annotations from GlymaID were differentially expressed between LG13-7552 (2n = 42) and LG12-7063 (2n = 40) and 397 of them had expression levels differences of at least fourfold between the two lines. Among those without a hit against a gene model, 69,525 (52%) contigs found matches in the soybean genome. Therefore, 98% of the assembled contigs matched sequences from the 40 soybean chromosomes of the reference Williams 82 genome.

RepeatMasker (RM) found that 4% of the Trinity contigs were retroelements (Supplemental Table 1). In the 26,126 contigs containing repetitive sequences, 1077 were significantly different in expression level between LG13-7552 (2n = 42) and LG12-7063 (2n = 40) (Table 2). Of these, 399 contigs were expressed in both lines and had higher abundance in the DAAL, compared to 242 higher in the 2n = 40 progeny. Gene expression of 342 contigs were unique to the DAAL and 94 were only expressed in the 2n = 40 progeny lines. Larger number of more abundant TEs and nearly four times the number of TEs were uniquely expressed in the DAAL as in the 2n = 40 line, indicating that TEs were less active in the 2n = 40 lines.

**Fig. 2:**
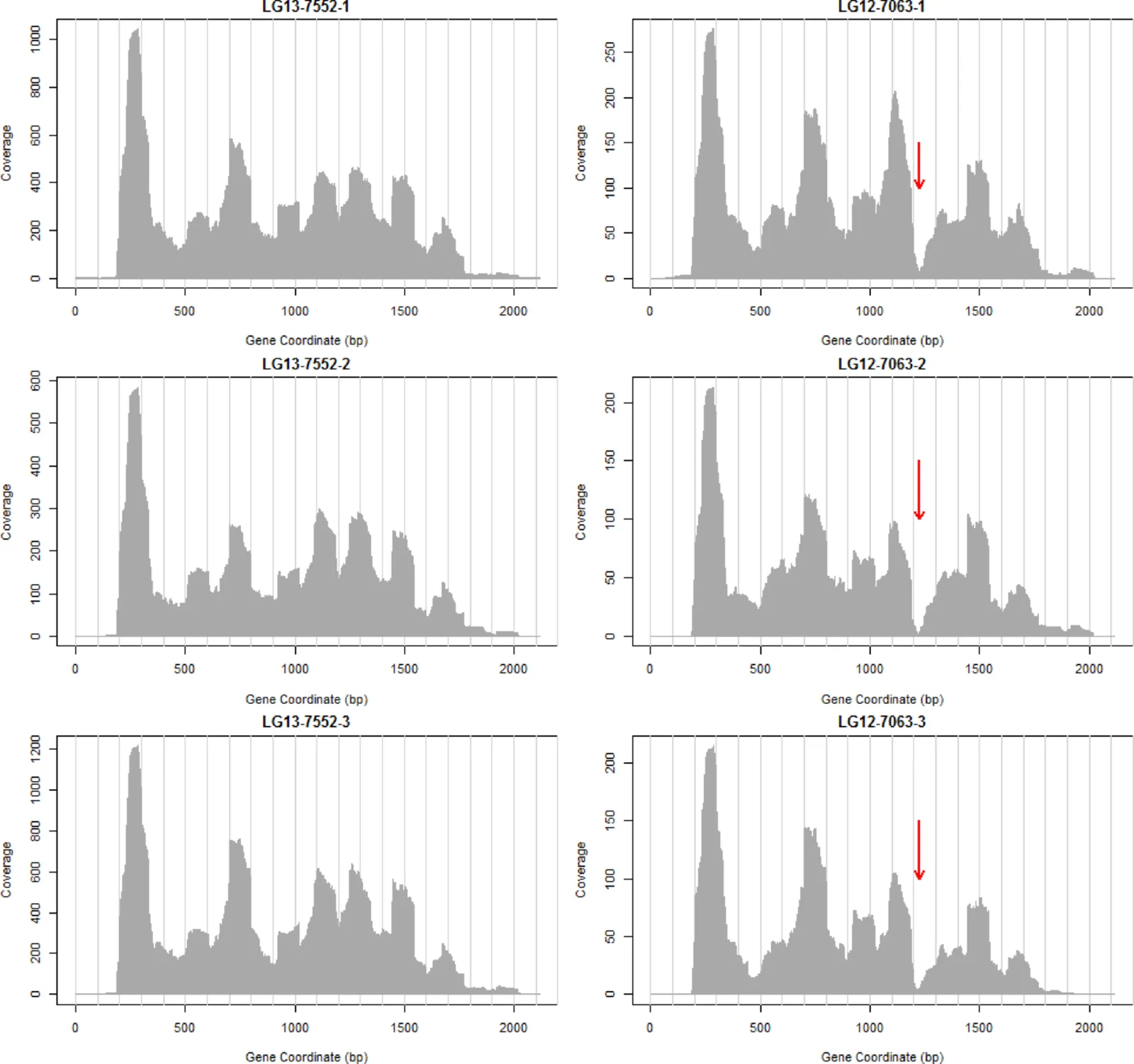
Distributions of RNA-seq reads of all three replicates of LG13-7552 and LG12-7063 that aligned to the gene contig c23278_g1 from Trinity de novo assembly. Contig c23278_g1 matched 100% to the* T* locus coding gene Glyma06g21920 from *G. max* Wm82.a2.v1. Each column of graphs represents the distribution of non-normalized RNA-seq reads from LG13-7552 and LG12-7063, from left to right, that aligned to the corresponding positions on the 2114 bp contig sequences from three replicates of each line are presented. Graphs are scaled to the maximum expression of each individual plant. The arrows in the right column mark the 1225 bp position of the 1 bp Adenine deletion in each LG12-7063 plant. The coverages of the sequence 50 bp upstream and downstream from the “A”-nt deletion were decreased when comparing to the expression patterns in the left column.

The Trinity assembled contig c23278_g1 had only one isoform and matched 100% to the *T* locus coding gene Glyma06g21920 sequence from *G. max* Wm82.a2.v1 (Phytozome, http://www.phytozome.net/) with one base pair difference compared to the nucleotide sequence of *G. max* sf3''''h1 mRNA for flavonoid 3''''-hydroxylase (GenBank: AB061212.1) sequence deposited in NCBI (http://www.ncbi.nlm.nih.gov) by Toda et al. (2002), which is a synonymous mutation. The transcription level of c23278_g1 in LG12-7063 was only a quarter of the level in the DAAL (Fig. 2), which was consistent with the phenotypic difference that LG12-7063 had gray pubescence while LG13-7552 was tawny. When comparing the distributions of RNA reads aligned to c23278_g1, a missing peak was found at the 1230 ± 50 bp region in LG12-7063. Part of the region had less than 5 mapped reads and at 1225 bp position all aligned reads are mismatches (Fig. 2). The structure of F3′H gene was reported to be a 8500 bp region contains three exons and two introns (Toda et al. 2005). The missing peak was located in the third exon but does not overlap with the previously reported position of the cytosine deletion, which was known as the *t* allele.

**Fig. 3:**
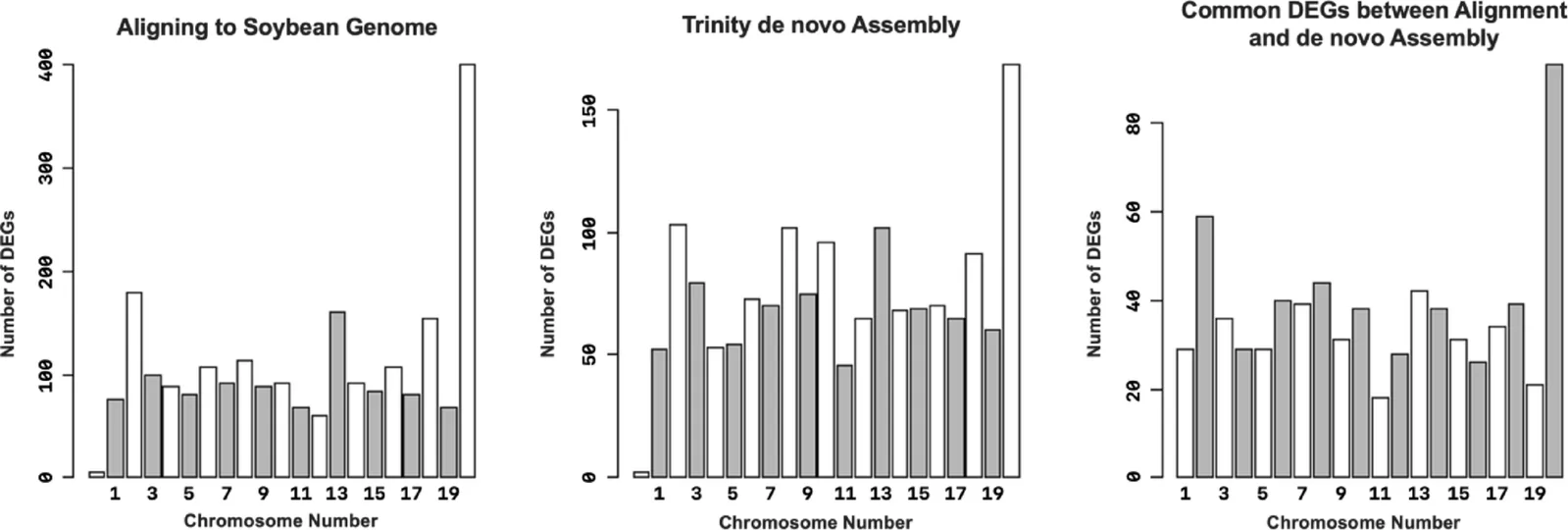
Distribution of differentially expressed genes (DEGs) between the DAAL (LG13-7552) and the 2n = 40 progeny (LG12-7063) on each chromosome from aligning RNA sequencing reads to the Williams 82 soybean reference genome (scaffold and 20 chromosomes), Trinity de novo assembly and common DEGs between the two methods. The x-axis indicates the chromosome number or ungrouped scaffolds (the bar to the left of chromosome 1). The y-axis indicates the number of DEGs located on each chromosome or ungrouped scaffolds.

DEGs identified from Novoalign aligning to the reference genome and Trinity de novo assembly were distributed across every soybean chromosome (Fig. 3). The majority of the two DEG sets were similarly distributed across all chromosomes with the largest number on chromosome 20, which indicates that differential expression was a global occurrence in the genome rather than chromosome/region specific. Among the 2292 DEGs from alignment and 1563 DEGs from de novo assembly, only 752 DEGs matched the exact same GlymaID and the distributions of the common DEGs also resembled distributions of the previous two DEG sets.

Although the statistical analysis of differential expressions in both methods (Trinity de novo assembly and Novoalign genome alignment) was conducted in DESeq, different abundance estimation tools were used to determine transcript expression level between the two methods. In the method by direct alignment of RNA-Seq reads to the reference genome, Novoalign uses a high sensitivity setting that encourages the mapping of more reads than other aligners. It also allows gaps in the alignment but lowers the MapQ of the alignment. The threshold of alignment scores was set to the default, which normally corresponds to alignments with 85% identity or better. In de novo assembly, the Trinity built-in abundance estimator RSEM and stringent alignment tool Bowtie promote higher accuracy in alignment. The mismatch parameter was set to 3 and gaps to zero. The use of different alignment methods is one of the main reasons why the number of DEGs from the Trinity de novo assembly was much less than that from direct alignment of RNA-Seq reads to the reference genome using Novoalign. Only about half of the DEGs from the Trinity analysis matched a GlymaID from the direct alignment analysis, which is also likely the result of mismatches with nearly identical paralogs, or due to de novo assembly of expressed sequences absent in the Williams 82 reference. To narrow down the analysis, only the 752 DEGs that were common to the two analysis methods were further processed. Most genes have definitive functional descriptions, but some, such as Glyma02g03280, Glyma11g27920, Glyma17g06540, Glyma07g07145, Glyma10g26990, Glyma10g39800 and Glyma05g36750 have no known functional annotation. Several stress and pathogen related plant defense genes were among the most differentially expressed genes between the DAAL (LG13-7552) and the 2n = 40 line (LG12-7063) (Tables 3 and 4). Glyma16g31341, Glyma17g31730, Glyma08g06730, Glyma03g16620, and Glyma18g29400 had lower expression in LG12-7063 than in LG13-7552; whereas Glyma01g05710, Glyma02g42315, Glyma02g10320, Glyma03g22060, Glyma03g03170, and Glyma11g00510 were higher in LG12-7063. Of these top defense DEGs, three are predicted to be leucine-rich repeat (LRR) receptor-like serine/threonine-protein kinases, which play central roles in signaling during pathogen recognition, the subsequent activation of plant defense mechanisms, and developmental control (Afzal et al. 2008).

**Table 3:** Functional annotations of top 20 most abundant genes in the DAAL (LG13-7552, 2n = 42) compared to the 2n = 40 progeny (LG13-7063, 2n = 40) from the common differentially expressed genes between aligning RNA sequencing reads to the soybean reference genome Williams 82 and Trinity de novo assembly. All the annotations are from Soybean Gene Expression Database (SGED).

GlymaID^a^	Annotations from NCBI BLAST^b^	Description^c^	Score^d^	E-value^e^	%ID^f^
Glyma20g33810	gi|356576401|ref|XP_003556320.1|	PREDICTED: anthocyanidin 3-O-glucosyltransferase-like [Glycine max]	941	0	100
Glyma16g31341	gi|356561629|ref|XP_003549083.1|	PREDICTED: LRR receptor-like serine/threonine-protein kinase GSO1-like [Glycine max]	2108	0	100
Glyma06g36140	gi|356514675|ref|XP_003526029.1|	PREDICTED: squamosa promoter-binding protein 1-like [Glycine max]	292	2E−95	100
Glyma02g13401	gi|356499925|ref|XP_003518786.1|	PREDICTED: MADS-box protein CMB1-like [Glycine max]	504	3E−175	100
Glyma17g31730	gi|356553315|ref|XP_003545002.1|	PREDICTED: 12-oxophytodienoate reductase 1-like [Glycine max]	585	0	77.1
Glyma17g08890	gi|356562644|ref|XP_003549579.1|	PREDICTED: agamous-like MADS-box protein AGL8-like [Glycine max]	490	2E−171	100
Glyma02g46311	gi|351722745|ref|NP_001235463.1|	uncharacterized protein LOC100527650 [Glycine max]	147	4E−41	99
Glyma14g37210	gi|356551857|ref|XP_003544289.1|	PREDICTED: dehydrodolichyl diphosphate synthase 2-like [Glycine max]	546	0	100
Glyma10g04230	gi|356536985|ref|XP_003537012.1|	PREDICTED: inorganic phosphate transporter 1–4-like [Glycine max]	1003	0	100
Glyma18g47090	gi|356566913|ref|XP_003551669.1|	PREDICTED: nucleolar protein 14-like [Glycine max]	1268	0	92.2
Glyma08g06730	gi|357476059|ref|XP_003608315.1|	Pathogenesis-related protein [Medicago truncatula]	450	8E−149	60.2
Glyma20g23220	gi|356575375|ref|XP_003555817.1|	PREDICTED: WUSCHEL-related homeobox 13-like [Glycine max]	577	0	100
Glyma03g16620	gi|357505745|ref|XP_003623161.1|	Protease inhibitor/seed storage/LTP family protein [Medicago truncatula]	179	1E−52	68
Glyma02g13420	gi|356499927|ref|XP_003518787.1|	PREDICTED: floral homeotic protein APETALA 1-like [Glycine max]	429	6E−147	100
Glyma20g30740	gi|225460644|ref|XP_002266350.1|	PREDICTED: thioredoxin Y1; chloroplastic [Vitis vinifera]	207	7E−62	79.3
Glyma18g29400	gi|356566177|ref|XP_003551311.1|	PREDICTED: AP2-like ethylene-responsive transcription factor PLT2-like [Glycine max]	908	0	100
Glyma17g06540	gi|356562052|ref|XP_003549289.1|	PREDICTED: uncharacterized protein LOC100793322 [Glycine max]	535	0	100
Glyma07g07145	gi|356520357|ref|XP_003528829.1|	PREDICTED: uncharacterized protein LOC100783381 [Glycine max]	698	0	87.4
Glyma18g53780	gi|356567298|ref|XP_003551858.1|	PREDICTED: probable methyltransferase PMT19-like [Glycine max]	1203	0	100
Glyma10g26990	gi|356533451|ref|XP_003535277.1|	PREDICTED: uncharacterized protein LOC100807304 [Glycine max]	691	0	100

^a^GlymaID identifier of differentially expressed genes (DEGs)

^b^Functional annotations of the DEGs from National Center for Biotechnology Information (NCBI) (blastx) database (2010)

^c^Descriptions of the functional annotations of the DEGs

^d^BLAST score to the alignment quality between the functional annotations and the DEGs

^e^The probability of the functional annotations and the DEGs sequences were matched by chance

^f^Percent identity between the functional annotations and the DEGs

**Table 4:** Functional annotations of top 20 most abundant genes in the 2n = 40 progeny (LG12-7063) compared to the DAAL (LG13-7552, 2n = 42) from the common differentially expressed genes between aligning RNA sequencing reads to the soybean reference genome Williams 82 and Trinity de novo assembly. All the annotations are from Soybean Gene Expression Database (SGED).

GlymaID^a^	Annotations from NCBI BLAST^b^	Description^c^	Score^d^	E-value^e^	%ID^f^
Glyma02g03280	gi|255640859|gb|ACU20712.1|	Unknown [Glycine max]	213	2E−63	100
Glyma05g25130	gi|356510754|ref|XP_003524099.1|	PREDICTED: reticuline oxidase-like protein-like [Glycine max]	825	0	78.1
Glyma01g05710	gi|357486941|ref|XP_003613758.1|	Disease resistance-like protein [Medicago truncatula]	1162	0	57.6
Glyma11g27920	No_hit				
Glyma02g42315	gi|356553790|ref|XP_003545235.1|	PREDICTED: probable LRR receptor-like serine/threonine-protein kinase At3g47570-like [Glycine max]	425	1E−137	65
Glyma02g10320	gi|356502432|ref|XP_003520023.1|	PREDICTED: heat shock cognate 70 kDa protein 2-like [Glycine max]	1201	0	100
Glyma06g16010	gi|356518775|ref|XP_003528053.1|	PREDICTED: ABC transporter G family member 5-like [Glycine max]	1264	0	100
Glyma03g22060	gi|356503056|ref|XP_003520328.1|	PREDICTED: TMV resistance protein N-like [Glycine max]	1343	0	97.3
Glyma04g38940	gi|356518777|ref|XP_003528054.1|	PREDICTED: pentatricopeptide repeat-containing protein At3g29230-like [Glycine max]	278	2E−85	91.9
Glyma06g43880	gi|356515120|ref|XP_003526249.1|	PREDICTED: UDP-glycosyltransferase 79B6-like [Glycine max]	929	0	100
Glyma04g04230	gi|83853828|gb|ABC47860.1|	N-hydroxycinnamoyl/benzoyltransferase 1 [Glycine max]	761	0	80.4
Glyma10g39800	gi|356577793|ref|XP_003557007.1|	PREDICTED: uncharacterized protein LOC100790784 [Glycine max]	95.5	1E−19	43.1
Glyma20g01470	gi|356577045|ref|XP_003556640.1|	PREDICTED: purple acid phosphatase 17-like [Glycine max]	653	0	100
Glyma03g03170	gi|356506370|ref|XP_003521957.1|	PREDICTED: probable LRR receptor-like serine/threonine-protein kinase At4g08850-like [Glycine max]	1447	0	100
Glyma19g01840	gi|356571919|ref|XP_003554118.1|	PREDICTED: cytochrome P450 82A4-like [Glycine max]	1087	0	100
Glyma05g36750	gi|351722649|ref|NP_001238275.1|	uncharacterized protein LOC100305633 [Glycine max]	277	4E−90	99.4
Glyma08g18033	gi|356528695|ref|XP_003532935.1|	PREDICTED: LOW QUALITY PROTEIN: 1-aminocyclopropane-1-carboxylate oxidase homolog 10-like [Glycine max]	634	0	99.7
Glyma07g08950	gi|356520493|ref|XP_003528896.1|	PREDICTED: gibberellin 20 oxidase 2-like [Glycine max]	830	0	100
Glyma11g00510	gi|356540317|ref|XP_003538636.1|	PREDICTED: cysteine-rich receptor-like protein kinase 10-like [Glycine max]	1151	0	100
Glyma02g40620	gi|356500976|ref|XP_003519306.1|	PREDICTED: medium-chain-fatty-acid–CoA ligase-like [Glycine max]	1111	0	100

^a^GlymaID identifier of differentially expressed genes (DEGs)

^b^Functional annotations of the DEGs from National Center for Biotechnology Information (NCBI)(blastx) database (2010)

^c^Descriptions of the functional annotations of the DEGs

^d^BLAST score to the alignment quality between the functional annotations and the DEGs

^e^The probability of the functional annotations and the DEGs sequences were matched by chance

^f^Percent identity between the functional annotations and the DEGs

Of the top 20 genes that had higher expression in the DAAL (Table 3), six are predicted to be genes involved in controlling a number of fundamental aspects of plant growth and development, such as meristem differentiation, shoot structure, flowering time, branching, and leaf initiation rate. These genes include Glyma06g36140, Glyma02g13401, Glyma17g08890, Glyma20g23220, Glyma02g13420, and Glyma20g30740. Two of these genes are predicted to be MADS-box proteins, which can be critical to gametophyte development, embryo and seed development, and root, flower and fruit development as well (Gramzow and Theissen 2010). The high ratio of differentially expressed critical genes that contribute to the fundamental plant growth captured in young DAAL seedlings suggests abnormal gene regulation of early plant development, which could explain curled and rugose trifoliolates of the DAALs and occasionally in misshaped leaflets or opposite growth position observed when harvesting tissue for sequencing. All six developmental genes identified are at least 16 times higher in expression in LG13-7552 than in LG12-7063; therefore, apart from the previous speculation of stress in the DAAL, overexpression of the critical genes involved in plant development could possibly be involved in the abnormal and stunted development of the DAAL as well. Except for the genes with ambiguous functional annotations and stress related genes, the remainder of the top 20 most abundant genes in the 2n = 40 progeny (Table 3) are involved in multiple pathways, such as ABC transporter, N-hydroxycinnamoyl/benzoyltransferase and purple acid phosphatase, which are difficult to associate with any of the phenotypic variation.

The gene that had largest expression difference was Glyma20g33810 (Table 3) was predicted to be an anthocyanidin 3-O-glucosyltransferase with 100% identity. Its expression was more than 512 times higher in DAAL lines than 2n = 40 progeny line. Anthocyanidin 3-O-glucosyltransferase catalyzes the reversible reaction of converting uridine diphosphate (UDP)-D-glucose and an anthocyanidin to UDP and an anthocyanidin-3-O-beta-glucoside (Kamsteeg et al. 1978). It was postulated to be one of the two key enzymes in the conversion from colorless leucoanthocyanidin to purple anthocyanidin 3-glucoside (Nakajima et al. 2001). This result is consistent with the phenotypic observation of DAALs possess purple hypocotyl and purple flower color, whereas, LG12-7063 has green hypocotyl and white flower colors.

### Presence of *G. tomentella* sequences in the DAAL and analysis of 2n = 40 lines

The functional annotations available from Phytozome (www.phytozome.net) are limited in the Williams 82 reference, and therefore 2752 (2% of total) of the Trinity assembled contigs had no annotation found in the soybean reference genome. When these unmatching contigs were compared (tblastx) to NCBI database, 1454 of them had high similarity to known sequences, and 213 of them matched *G. tomentella* sequences with percent identities ranging from 37 to 100% (Table 2). Of the 213 contigs, 20 had homologous protein matches (E-value < 1e−06) from NCBI database (blastx) and are listed in Supplemental Table 2.

These 213 assembled contigs that matched *G. tomentella* sequences were grouped into 175 genes that matched 22 publicly deposited *G. tomentella* sequences, which were made up of 10 BAC- clones and 12 retrotransposon sequences. Of the 175 matched genes, 174 were expressed only in the DAAL and 51 of the genes had statistically significant differential expression between the two lines (p_adj_ < 0.05). This confirms that part of the *G. tomentella* genome was present and expressed in the DAAL and are likely located in the extra pair of *G. tomentella* chromosome. It is possible they have transposed into the 40 soybean chromosomes but if they were, we would expect to detect the expression of at least some of them in the 2n = 40 line. All 22 sequences that showed *G. tomentella* as the best database match were annotated as either BAC clones or transposable elements (TEs) and no additional details regarding their functions are available. It is possible that some of these represent uncharacterized TEs that moved into the soybean chromosomes of the 2n = 40 line.

One of the 213 contigs that matched *G. tomentella* sequences had reads mapped to it from both lines and two were expressed only in the 2n = 40 progeny; however, all of these three had very low read counts (< 10) and none of them had significantly different expression levels, which means they are likely to be false positive read counts. Therefore, we found no evidence of expressed *G. tomentella* sequences in the 2n = 40 progeny.

There are few *G. tomentella* sequences available in the database, so those that match *G. tomentella* sequences are not likely to be all of the introgressed *G. tomentella* genes. Many of the contigs that did not match the soybean reference matched sequences from other legume species, such as *Medicago trunculata* Gaertn. It is possible that these sequences could be genes from *G. tomentella* that are highly conserved across species in the Fabaceae.

The TEs detected in the DAAL likely originated from the additional two *G. tomentella* chromosomes. In the first use of RM, total assembled contigs were blasted against the *Glycine* repetitive element database, but few TEs were found. Since the 4% retro-elements were found using a BLAST search against all the eudicotyledons but not when using *Glycine* sequences, they are unlikely to be soybean sequences. The 12 matched *G. tomentella* retrotransposon sequences from NCBI database support this idea, as well as RM confirming that 56 of the 175 genes that matched *G. tomentella* sequences deposited in NCBI were retrotransposons. These uniquely expressed TEs in the DAAL are most likely located on the two *G. tomentella* chromosomes.

Since only RNA was sequenced in this study, the genomic location or effect of DNA transposons on the phenotype cannot be determined. The SoyTEdb shows that 42% of the soybean genome consists of class I TEs (retrotransposons), whereas class II TEs (DNA transposons) account for 16% of the soybean genome (Du et al. 2010a). Class I TEs involve an RNA intermediate when inserted at a new position, but class II does not. Although DNA transposons are less common in the genome, they tend to disproportionately insert into or near genes and thus are more likely to trigger phenotypic differences.

### A novel isoform of a recessive allele at the *T* locus

**Fig. 4:**
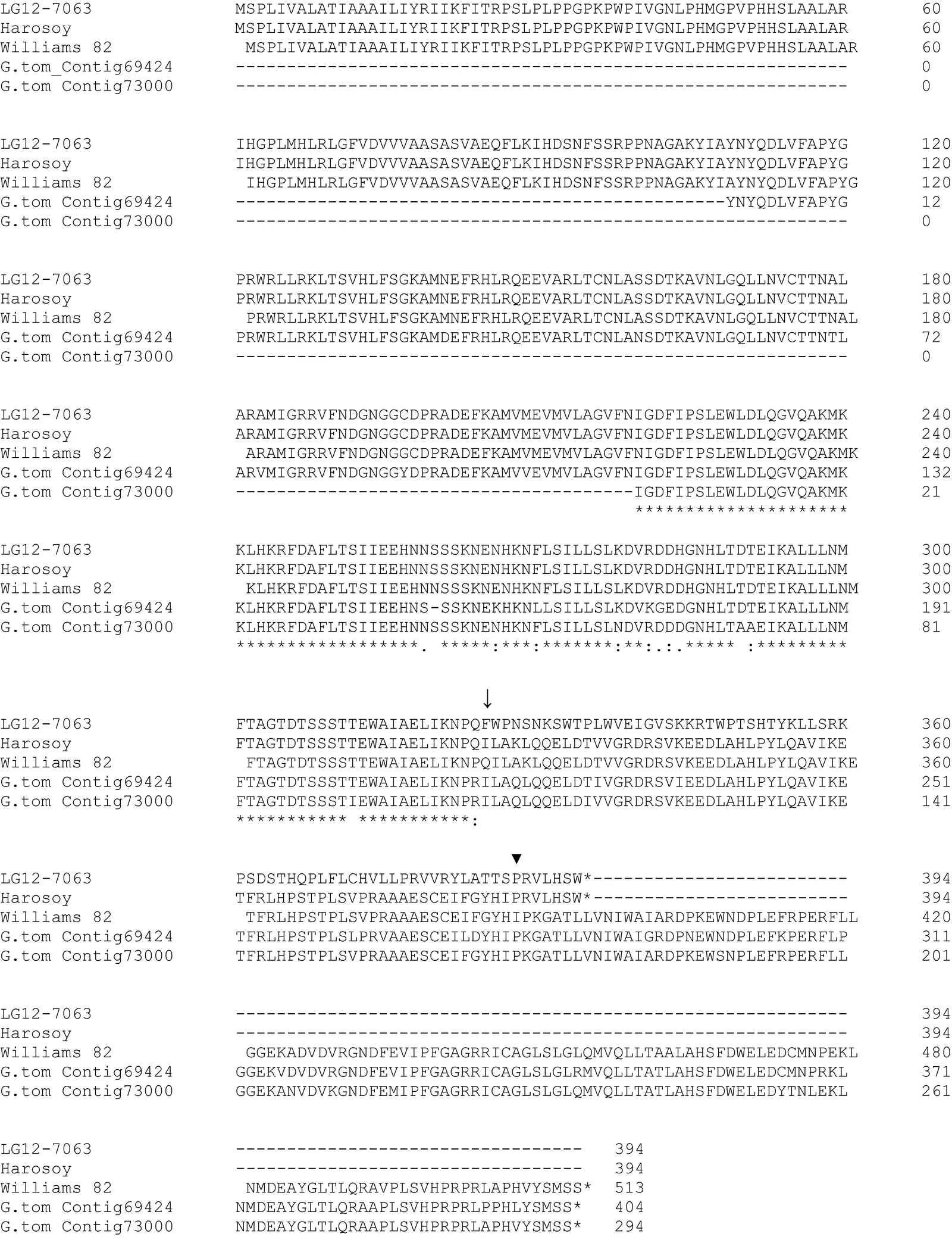
Flavonoid 3′ hydroxylase amino acid sequences alignment of LG12-7063 (*t-a*), Harosoy (*t*), Williams 82 (*T*) and two *G. tomentella* transcripts. The compared amino acid sequences are translated from LG12-7063 genomic DNA, Harosoy (GenBank accession no. AF499734) and Williams 82 (GenBank accession no. AB061212.1) cDNAs. The Phenylalanine (F) at position 325 in LG12-7063 (noted by arrow) resulted from an “A”-nt deletion that shifted the open reading frame of the rest of the gene. The proline (P) at position 388 in Harosoy (indicated by an arrowhead) was from a “C”-nt deletion that as well shifted the frame. Both frameshifts lead to the early termination of the protein translation and the shortage of 124 amino acids at the 3′ end of the polypeptide (represented by dash).

DNA sequencing confirmed LG12-7063 had a single base adenine (A) deletion in the exon 3 of flavonoid 3′ hydroxylase gene, which explains the gray pubescence and buff hilum color. This “A” deletion shifted the reading frame that converted the subsequent amino acid from isoleucine to phenylalanine (ATT to TTT) and introduced a premature stop codon 34 amino acids downstream, This resulted in a truncated polypeptide that is lacking 124 amino acids at the 3′ end (Fig. 4). This truncation is similar to the *t* allele of soybean cultivars Richland and Harosoy (Zabala and Vodkin 2003) as they terminate at same amino acid position, although other amino acid residues within the gene are different to the those in the target line described here. F3′H belongs to the cytochrome P450 protein family, which determines its hydroxylation patterns of the B-ring to the flavonoid substrates (Stafford 1983; Kaltenbach et al. 1999). The conserved amino acid sequence motif (FXXGXXXCXG) for the heme-binding domain in the C-terminal of the polypeptide is missing due to the truncation in LG12-7063 (Wachenfeldt and Johnson 1995). The transcription level of c23278_g1 in LG12-7063 was only a quarter of the level in the DAAL, which suggested that the transcribed RNA in LG12-7063 was undergoing nonsense-mediated mRNA decay because of the premature stop codon (Baker and Parker 2004).

This “A” deletion within the *T* locus has not been previously documented. A search of the *G. tomentella* transcriptome revealed that this deletion is not present in the perennial parent (Fig. 4). We were not able to find a *G. max* accession with the same sequence at this locus, yet screening of a larger sample of *G. max* germplasm may identify a line in which the derived line haplotype exists.

### Phenotypic variation among the DAAL-derived progeny populations

Field data from 2013 and 2014 demonstrated significant differences among DAAL-derived lines for all quantitative traits measured. Lines with either protein concentrations higher or lower than Dwight were identified (Table 1). No line had higher oil concentration than Dwight but several were lower (Table 1). There were lines that were either taller or shorter than Dwight, but there were only lines that lodged more or were later in maturity than Dwight (Table 1). In both experiments the DAALs were similar in morphology and phenotypes (Table 1).

Several qualitative traits that were not present in either parent or in the progenitor DAAL were also documented (Table 5). LG15-30921-1 had appressed pubescence, which indicates this line may carry an uncommon allele in modern soybean, *pa1* (Bernard 1975). Deciduous pubescence was found in LG15-30865-1, LG15-30899-1, LG13-30906-3, and LG15-30921-4 and is controlled by the *pc* allele in *G. max* (Bernard and Singh 1969). It is an extremely rare trait occurring in only 0.3% of the soybean accessions in the USDA Soybean Germplasm Collection. LG15-30780-1 segregated for sterility; however, its causal factor is unknown since multiple mechanisms can result in sterility including nine genes related to male sterility (Brim and Young 1971; Boerma and Cooper 1978; Palmer et al. 1980; Delannay and Palmer 1982; Buss 1983; Graybosch et al. 1984; Skorupska and Palmer 1989; Palmer 2000), seven synaptic mutations (Hadley and Starnes 1964; Palmer 1974; Palmer and Kaul 1983; Ilarslan et al. 1997; Palmer and Horner 2000), and several cytoplasmic male-sterile systems (Sun et al. 1997; Palmer et al. 2001; Smith et al. 2002). LG15-30536-1 shattered extensively at maturity, which was a key agronomical trait that was intensively selected against during soybean domestication (Swarm et al. 2019).

**Table 5:** Summary of qualitative traits among the disomic alien addition line (DAAL)-derived 2n = 40 progeny lines in 2016 replicated field study.

Trait	Dwight and DAAL (2n = 42)	*G. tomentella* PI 441001	Derived progenies (2n = 40)
Pubescence	Tawny	Tawny	Tawny, Gray, Light tawny
Pubescence Type	Erect	Erect	Erect, Semi-appressed, Appressed, Deciduous
Seed Coat Color	Yellow	Black	Yellow, Black, Imperfect black, Buff, Green, Reddish brown
Hilum Color	Black	Black	Gray, Yellow, Black, Imperfect black, Brown, Buff, Reddish brown
Flower Color	Purple	Purple	Purple, White
Pod Color	Brown	Black	Brown, Tan, Black
Stem Termination	Indeterminate	Perennial	Determinate, Indeterminate

Chromosomes were counted for 108 progenies that exhibited different phenotypes from DAALs and all were confirmed to have 40 chromosomes. Based on these data, we used phenotypic changes from the typical DAAL morphology as an indication of reverted soybean progeny. To conclusively show the presence of *G. tomentella* chromosomes in these derived lines and their subsequent generational loss, we made genomic in situ hybridization (GISH) probes using Fluorescein labelled *G. tomentella* genomic DNA and Fluorescent in situ hybridization (FISH) probes using Texas Red labelled oligos to *G. max* centromeric repeats. The GISH probes label *G. tomentella* whole chromosomes and the FISH probes label all *G. max* centromeres (Supplemental Fig. 2). In the derived lines, the FISH/GISH showed that the DAAL LG13-7552 had 40 *G. max* and 2 *G. tomentella* chromosomes. LG12-7063 had only 40 chromosomes that were all *G. max*, showing that it had lost the 2 *G. tomentella* chromosomes (Fig. 1). The GISH probes exhibited a cross-hybridization to the Nucleolar Organizer Region (NOR) in *G. max* that contains rDNA genes. This NOR signal is seen in LG13-7552, LG12-7063 and the recurrent parent Dwight (arrow heads in Fig. 1 and Supplemental Fig. 2).

Field studies from 2013 to 2016 showed that the frequency of DAAL progeny losing the *G. tomentella* chromosomes varied from 3 to 9% with an average of 4% (Table 6). The characteristics of the derived soybean progenies (2n = 40) were highly variable. Some lines partially resembled Dwight, but others were phenotypically different from either of the parents and their progenitor DAAL for many traits.

**Table 6:** Field observations of the DAALs from 2013 to 2016. Seeds from single DAAL plants were planted in rows and documented for the number of deviant plants within each row.

	No. of rows derived from single plants	No. of total deviant plants	Percentage^a^
2013	131	72	3
2014	30	48	9
2015	36	23	4
2016	28	18	4
Total	225	161	4

^a^The percentage is calculated as the number of the deviant plants divided by the total number of mature plants

### Genetic variation for specific loci

To document the genetics of phenotypic variation at a single trait level, three loci that control pubescence color, stem termination or seed coat color were sequenced. Sequencing the F3′H fragments (Toda et al. 2005) confirmed the DAAL (LG13-7552) and parent Dwight have identical sequences to the Williams 82 T allele. LG12-7063 had a single base adenine deletion at the 1225 bp position in c23278_g1, which is 204 bp upstream from the location of the cytosine deletion in the *t* allele reported by Toda et al. (2002). Among the selected soybean progenies, the majority of the gray pubescent lines such as LG15-30473-3 and LG15-30360-6 had the reported cytosine deletion. Lines, such as LG12-7063 and LG12-7086, had a variant allele, a single base adenine deletion in Exon3. *G. tomentella* has tawny pubescence and we identified 2 distinct transcripts from its transcriptome. At the nucleotide in question, both had no deletion and the amino acid residue was identical to Williams 82 and had no premature stop (Fig. 4).

The *Dt1* locus that controls stem termination in soybean is the *GmTfl1* gene, which is considered to be a homolog of a regulatory gene of a signaling protein in shoot meristem, terminal flower 1 in *Arabidopsis* (Alvarez et al. 1992; Bradley et al. 1997; Tian et al. 2010). There are four SNPs, each an independent form of the recessive *dt1* allele, designated as *GmTfl1-ta, GmTfl1-tb, GmTfl1-ab* and *GmTfl1-bb.* No known determinant accession contains more than one of the four SNPs. Dwight, PI 441001, and the DAAL had the same genotype as the Williams 82 reference for the *GmTfl1* region (Table 7). Among the derived soybean progeny population, three of the four recessive alleles of *Dt1* locus were found, *GmTfl1-ab, GmTfl1-tb,* and *GmTfl1-bb* (Table 7).

**Table 7:** SNP variants in the *Dt1* locus among references and the DAAL-derived 2n = 40 progenies.

Entry	Phenotype	Genotype			
		GmTfl1-ta	GmTfl1-ab	GmTfl1-tb	GmTfl1-bb
Reference					
PI 441001	Perennial	G	C	G	A
CNS	Determinate	G	T	G	A
Ogden	Determinate	G	C	G	T
Peking	Determinate	T	C	G	A
Soysota	Determinate	G	C	A	A
Dwight	Indeterminate	G	C	G	A
DAAL	Indeterminate	G	C	G	A
DAAL-derived Progeny (2n = 40)					
LG12-7063	Determinate	G	C	A	A
LG15-30139-5	Determinate	G	T	G	A
LG15-30253-1	Determinate	G	C	G	T

**Fig. 5:**
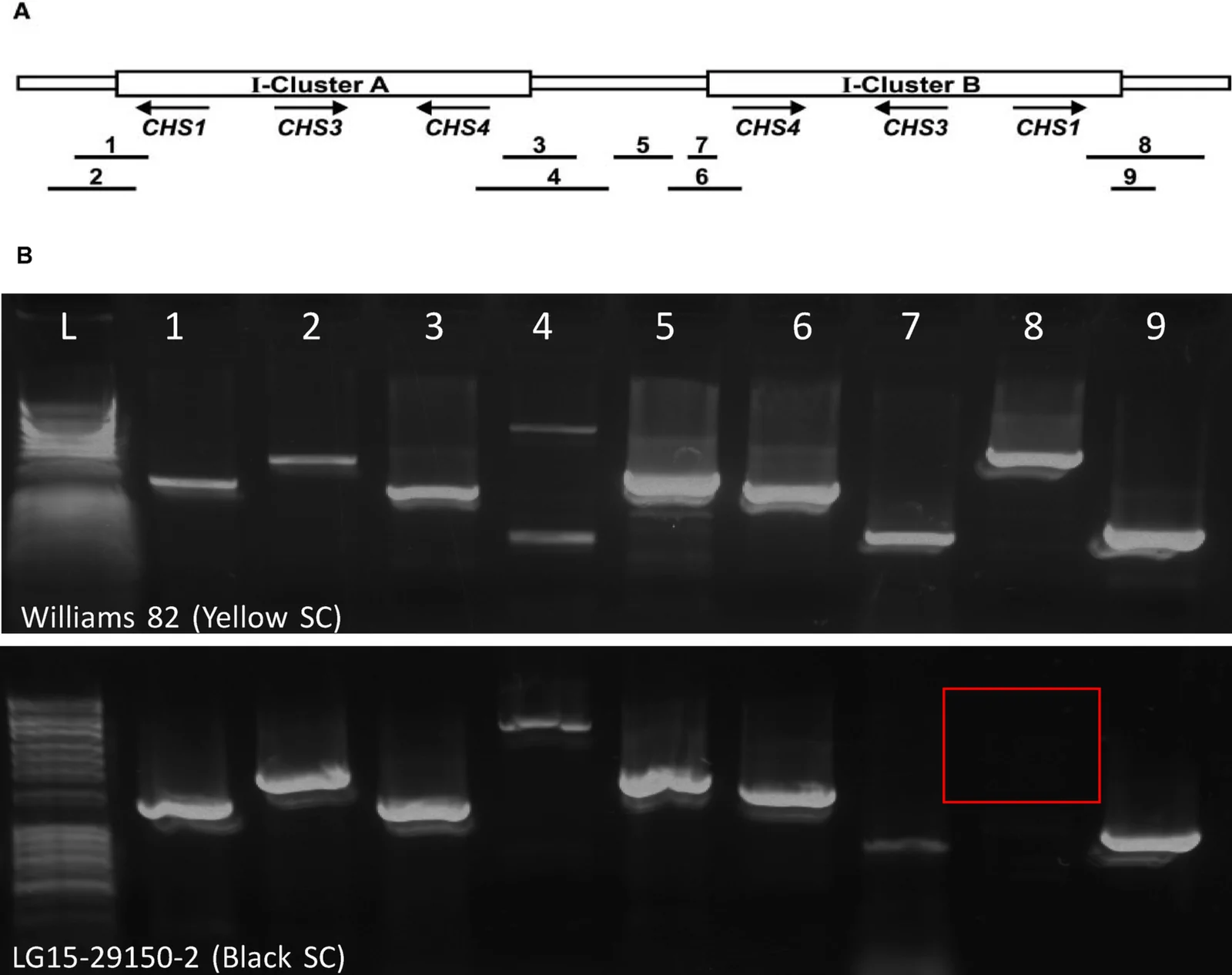
PCR verification of *I* locus. **A** The structure of two perfect inverted repeats of chalcone synthase (CHS) genes in the *I* locus of yellow seed coat soybean (Clough et al. 2004). The underlines signify the location of the amplifying fragments in the PCR verifications of the inverted repeats structure. **B** The PCR result of Williams 82 and LG15-29150-2 to verify the inverted repeat structure on the *I* locus. The number on the gel bands corresponds to the fragment locations in A, and L stands for ladder.

Two alleles, *I* and *i*^*i*^, at the *I* locus that control seed pigmentation, harbor two perfect inverted repeats of chalcone synthase (CHS) gene clusters (CHS1, CHS3, and CHS4) that enable the production of small RNAs and silencing all CHS genes in the seed coat (Fig. 5A) (Tuteja et al. 2004, 2009; Clough et al. 2004). In the PCR verification assay, all nine DNA regions amplified from the soybean parent Dwight and the progenitor DAAL matched the pattern from the yellow-seed coat *i*^*i*^ reference Williams 82 (Fig. 5B) which was expected as both have yellow seed coats. PCR failed to generate clear bands in most of the regions from PI 441001, which possibly indicates a different structure of CHS genes in the PI 441001 genome than in Dwight and the DAALs. Related black and yellow seed coat lines were examined to determine the structural differences. The yellow soybean lines derived from the DAAL had a complete set of nine PCR products; however, in the black seeded lines fragment #8 failed to amplify, for example LG15-29150-2.

### Genome-wide variation among the derived progenies

After filtering for minor allele frequencies and missing data, 22,360 SNPs were retained from genotyping-by-sequencing for genetic distance calculation among a wide range of DAAL-derived soybean progenies. The distances between 124 revertant progenies and Dwight ranged from 0.13 to 0.35 with a mean of 0.26, and 86% of them had genetic distances of at least 0.2 from Dwight. The distances between the progenies and the DAAL were from 0.07 to 0.33 with a mean of 0.23. Among the progenies, 37 lines (30%) were less than 0.2 genetic distance from the DAAL. These data show that most revertant progenies were different from Dwight and the progenitor DAAL. Among the 124 soybean progenies, DAAL and Dwight, genetic distances ranged from 0.05 (LG15-29872-1 and LG15-29905-2) to 0.44 (LG15-29464-4 and LG15-29473-1). While 86% (13,560 out of 15,750) of the pairs have genetic distances between 0.2 and 0.35, 9% of the pairs had genetic distances of 0.35 or greater. The differences among lines were found on all 20 pairs of chromosomes.

The genome wide similarity analysis using the genotype data from Illumina Soy6KSNP Beadchip array found genetic distances between the DAALs and the derived revertant soybean progenies varied from 0.01 to 0.13, while the genetic distances within the DAALs varied from 0.01 to 0.02. Of the six thousand SNP markers, eleven were not represented in this population, but none of the other markers were missing from more than 50% of the samples genotyped. The genetic distances of the 2n = 40 progenies ranged from 0.15 to 0.30 to Dwight, and 0.28 to 0.37 to Williams 82, which showed the derived soybean progenies more closely related to their progenitor DAAL than to either Dwight or Williams 82.

**Fig. 6:**
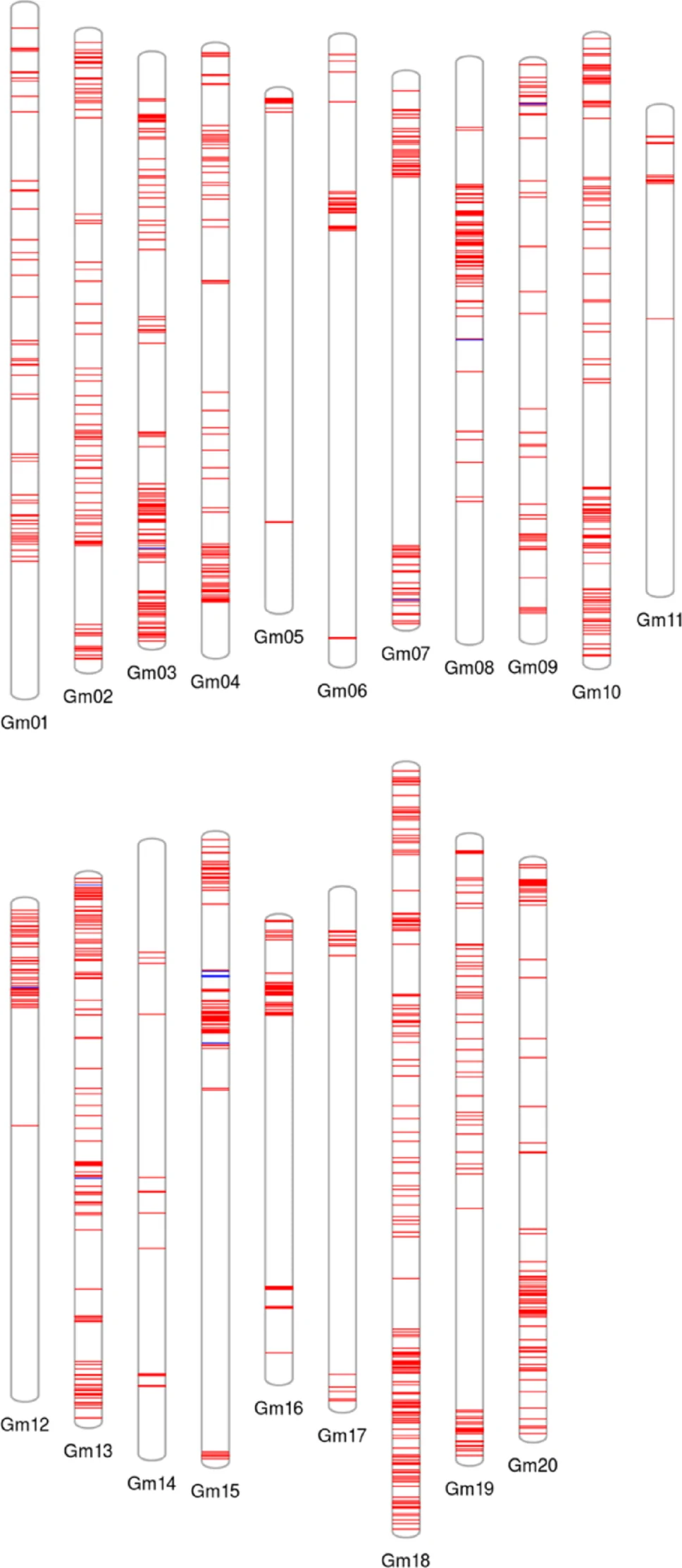
Physical locations of the polymorphic SNPs between LG15-30695-1, an off-type progeny, and LG15-30695-2, a DAAL sister line used as a reference. Heterozygous SNPs in the reference were excluded from analysis. Each line marks the physical location of a polymorphic SNP in the soybean genome. Red lines indicate heterozygous SNPs in the deviant and blue lines indicate homozygous SNPs.

LG15-30695-2 was a DAAL plant that was highly homozygous. The sister soybean line, LG15-30695-1, was a reverted progeny from the DAAL LG15-30695. LG15-30695-1 was described as having a lighter pod color and greater pod set than the DAAL in the same row. The genetic distance between LG15-30695-1 and LG15-30695-2, a typical DAAL plant, from the same row, was 0.11 with changes detected on all twenty pairs of chromosomes (Fig. 6). Most polymorphic markers were heterozygous in LG15-30695-1, except for 11 SNPs located on chromosome 3, 9, 13, and 15 (Fig. 6). With only minor phenotypic differences observed between LG15-30695-2 and LG15-30695-1, there were still many genetic differences distributed throughout the genome.

### Field observations of traits of interests among 2n = 40 progenies

While documenting the wide phenotypic variation among the 2n = 40 progenies derived from the DAALs, “rogue plants” were noticed in some plant rows. These plants appeared to be the same as all other plants in the row except for changes in either seed coat color, pubescence color or stem termination. Plants with yellow seed coats were found in rows derived from a plant with black seed coat seeds in the previous year, tawny pubescent progeny from a gray pubescent plant, and indeterminate plants emerged from progeny from a determinate plant. All of the deviant phenotypes are normally conditioned by dominant alleles and would be very rare mutations. The frequency of the phenotypic changes over three years (only two years for stem termination) averaged 0.7% for the three traits of interest (Table 8). This is substantially lower than the rate of appearance of all revertant plants reported in Table 6. From 2014 to 2016, the number of plants with yellow seed coats found in rows planted with seeds from plants with black seedcoats ranged from 26 to 43, a change rate of 0.3% to 1.1%. The range is from 0.4% to 1.3% for pubescence color, and only 0.2% for year 2015 and 2016 in stem termination.

**Table 8:** Field observations on the DAAL-derived 2n = 40 progenies for three traits. Seeds from single plants with one of the recessive traits, black seed coat or gray pubescence, were planted in rows in independent blocks from 2014 to 2016. Plant rows with determinate growth type were observed from 2015 to 2016. Each single plant row was documented for the number of plants of each phenotype of interest.

Seed Coat Color				Pubescence Color				Stem Termination		
	2014	2015	2016		2014	2015	2016		2015	2016
No. of plant rows with black seed coat	131	213	68	No. of plant rows with gray pubescence	65	111	437	No. of determinate plant rows	82	140
No. of plants with yellow seed coats	43	26	31	No. of plants with tawny pubescence	23	47	66	No. of indeterminate plants	6	9
No. of plants with black seed coats	3961	8500	2715	No. of gray plants with gray pubescence	1699	4360	18,354	No. of determinate plants	2468	5600
Percentage^a^	1.10%	0.30%	1.10%	Percentage	1.30%	1.10%	0.40%	Percentage	0.20%	0.20%

^a^The percentage is calculated as the number of deviant plants with the dominant phenotype divided by the number of mature plants

Segregation ratios in the progeny of the deviant plants with dominant traits derived from a recessive progenitor were counted in the following generation. The segregation ratio of progenies from tawny pubescence deviants and indeterminate deviants fit the expected Mendelian ratio of 3:1 with only one exception when slightly more tawny plants than expected were counted. However, several of the progenies from yellow seed coat deviants did not segregate as anticipated. In 2014, seven yellow seed coat deviants harvested from black seed coat rows in 2013 were planted in the field. Only three out of seven rows (LG14-10063, LG14-10096, and LG14-10111) segregated in 3:1 yellow:black ratio (Table 9). One line (LG14-10014) had all black seed coat plants, another line (LG14-10166) had all yellow seed coat plants, and two rows (LG14-10051 and LG15-29087) had mostly black seed coat plants (Table 9). In 2015, Six out of thirteen yellow seeded deviants did not segregate 3:1 (Table 9). Again, one line (LG15-29111) had only yellow seed coat plants, and two had nearly twice as many black coat plants as expected. One line had only black seed coat plants and two lines fit a 3:1 ratio except the presumed recessive phenotype, black seed coat, was the majority trait. Four out of fifteen planted in 2016 didn’t fit the Mendelian ratio. In two instances, there were insufficient black seed coat plants and in two instances there were insufficient yellow seed coat lines (Table 9).

**Table 9:** Segregation ratios within populations derived from yellow seed coat deviants found in rows planted with seeds from plants with black seed coats.

Year	Field row	Seed source	Observed no. of dominant phenotype (yellow seed coat)	Observed no. of recessive phenotype (black seed coat)	Expected no. of dominant phenotype	Expected no. of recessive phenotype	*p* value from chi- square test
2014	LG14-10014	LG13-7355-9	0	9	7	2	0.00
2014	LG14-10051	LG13-7360-19	1	12	10	3	0.00
2014	LG14-10063	LG13-7384-9	24	8	24	8	1.00
2014	LG14-10096	LG13-7426-23	40	12	39	13	0.75
2014	LG14-10111	LG13-7427-6	33	11	33	11	1.00
2014	LG14-10154	LG13-7453-10	11	32	32	11	0.00
2014	LG14-10166	LG13-7453-22	19	0	14	5	0.01
2015	LG15-29087	LG14-10009-38	3	13	12	4	0.00
2015	LG15-29111	LG14-10010-24	40	0	30	10	0.00
2015	LG15-29112	LG14-10010-25	9	2	8	3	0.60
2015	LG15-29142	LG14-10011-29	32	11	32	11	0.93
2015	LG15-29190	LG14-10012-42	6	16	17	6	0.00
2015	LG15-29235	LG14-10054-6	34	13	35	12	0.67
2015	LG15-29237	LG14-10054-8	0	40	30	10	0.00
2015	LG15-29244	LG14-10054-15	19	6	19	6	0.91
2015	LG15-29254	LG14-10054-25	36	10	35	12	0.61
2015	LG15-29281	LG14-10107-13	22	15	28	9	0.03
2015	LG15-29282	LG14-10107-14	23	17	30	10	0.01
2015	LG15-29293	LG14-10107-25	27	9	27	9	1.00
2015	LG15-29314	LG14-10107-46	4	1	4	1	0.80
2016	LG16-7256	LG15-29052-1	35	3	29	10	0.01
2016	LG16-7276	LG15-29061-1	11	17	21	7	0.00
2016	LG16-7296	LG15-29068-1	39	8	35	12	0.21
2016	LG16-7316	LG15-29074-1	36	6	32	11	0.11
2016	LG16-7336	LG15-29078-1	29	11	30	10	0.72
2016	LG16-7370	LG15-29090-1	30	11	31	10	0.79
2016	LG16-7391	LG15-29092-1	25	8	25	8	0.92
2016	LG16-7392	LG15-29092-2	32	5	28	9	0.11
2016	LG16-7403	LG15-29092-13	25	10	26	9	0.63
2016	LG16-7410	LG15-29092-20	22	8	23	8	0.83
2016	LG16-7433	LG15-29111-3	28	9	28	9	0.92
2016	LG16-7451	LG15-29116-1	24	6	23	8	0.53
2016	LG16-7587	LG15-29158-11	0	5	4	1	0.00
2016	LG16-7597	LG15-29273-1	25	9	26	9	0.84
2016	LG16-7617	LG15-29277-1	29	2	23	8	0.02

**Fig. 7:**
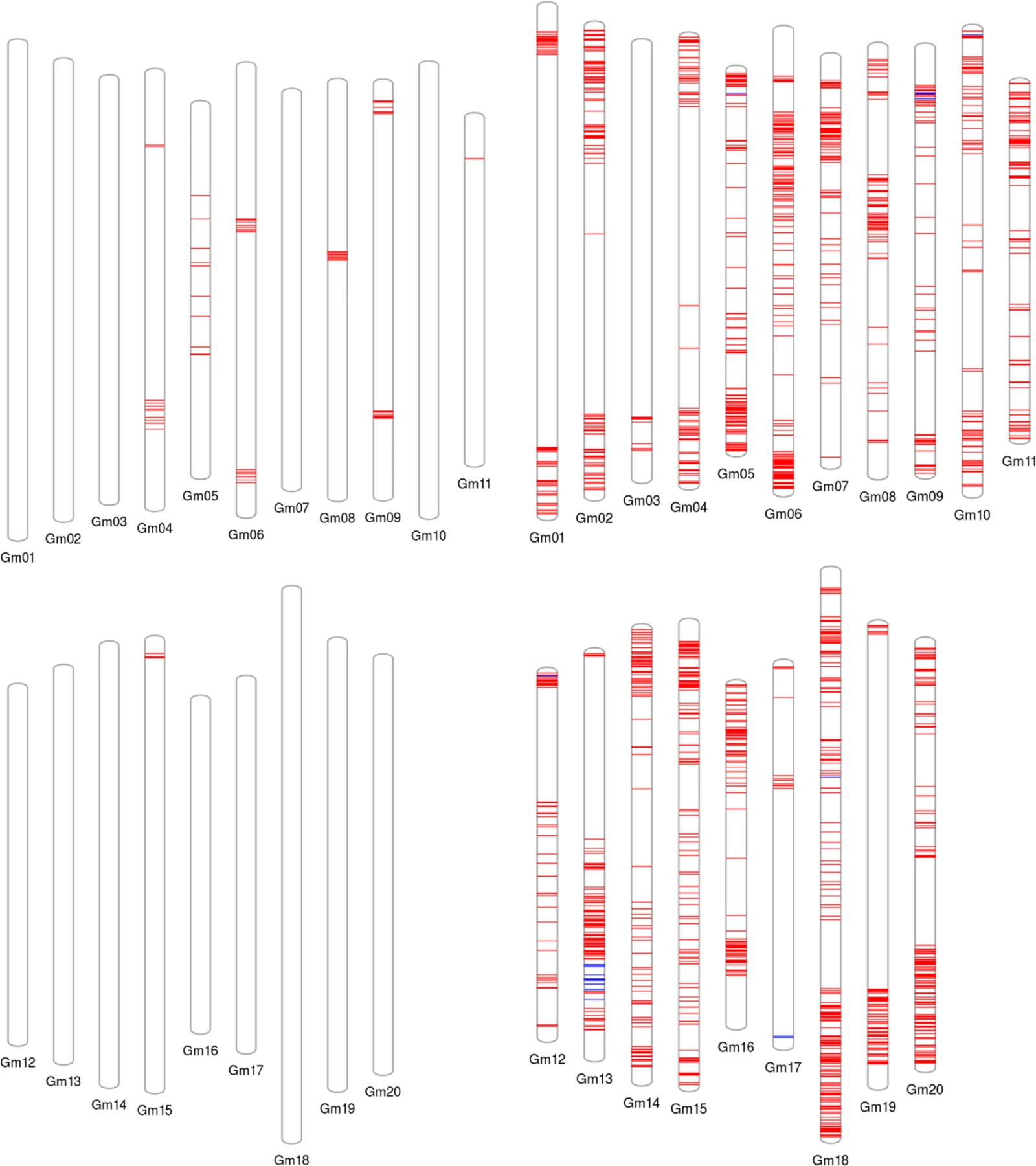
Physical locations of the polymorphic SNPs between LG15-30473-1, a tawny pubescence deviant, and LG15-30473-3, a gray pubescence sister line of the deviant used as a reference (left), and between LG15-30899-5, an indeterminate deviant, and LG15-30899-1, a determinate sister line of the deviant used as a reference (right). Heterozygous SNPs in the reference were excluded from analysis. Each line marks the physical location of a polymorphic SNP in the soybean genome. Red lines indicate heterozygous SNPs in the deviant and blue lines indicate homozygous SNPs.

The genome wide similarity analysis between twelve pairs of deviants, selected for changes in seed coat or pubescence color or stem termination, and corresponding sister lines using the genotypic data from Illumina Soy6KSNP beadchip array found genetic distances that varied from 0.01 to 0.15. Of the six thousand SNP markers, eleven SNP markers were completely missing from this population, but none of the rest of markers were missing from 50% or more of the samples genotyped. Comparing the 2n = 40 progenies lines with the parent Dwight and an elite soybean Williams 82, the genetic distances of the 2n = 40 progenies were from 0.15 to 0.3 to Dwight, and 0.28 to 0.37 to Williams 82. The reference sister lines with the recessive phenotype of trait of interest had minimal heterozygosity compared to the deviant. The number of heterozygous markers varied among deviants but most of the markers were heterozygous. The polymorphisms were spread across all twenty soybean chromosomes. For example, between LG15-30473-1, a tawny pubescence deviant found in a gray pubescence row, and LG15-30473-3, its gray pubescence sister line, the genetic distance was 0.01 and the two were indistinguishable except for pubescence color (Fig. 7). LG15-30899-5 was the indeterminate progeny from a determinate progenitor, LG165-30899-1 (Fig. 7). The genetic distance between these two lines was 0.15 but in comparing the genotypes of these two lines, polymorphic SNPs were found on all 20 chromosomes.

## Discussion

### Gene ontology enrichment analysis

Gene Ontology (GO) provides a controlled language to describe the attributes of genes and gene products associated with biological processes, cellular components, and molecular functions. Among the differentially expressed genes from the alignment analysis, 1529 have GO annotations. Their GO terms enrichments were compared with the reference Williams 82.

**Fig. 8:**
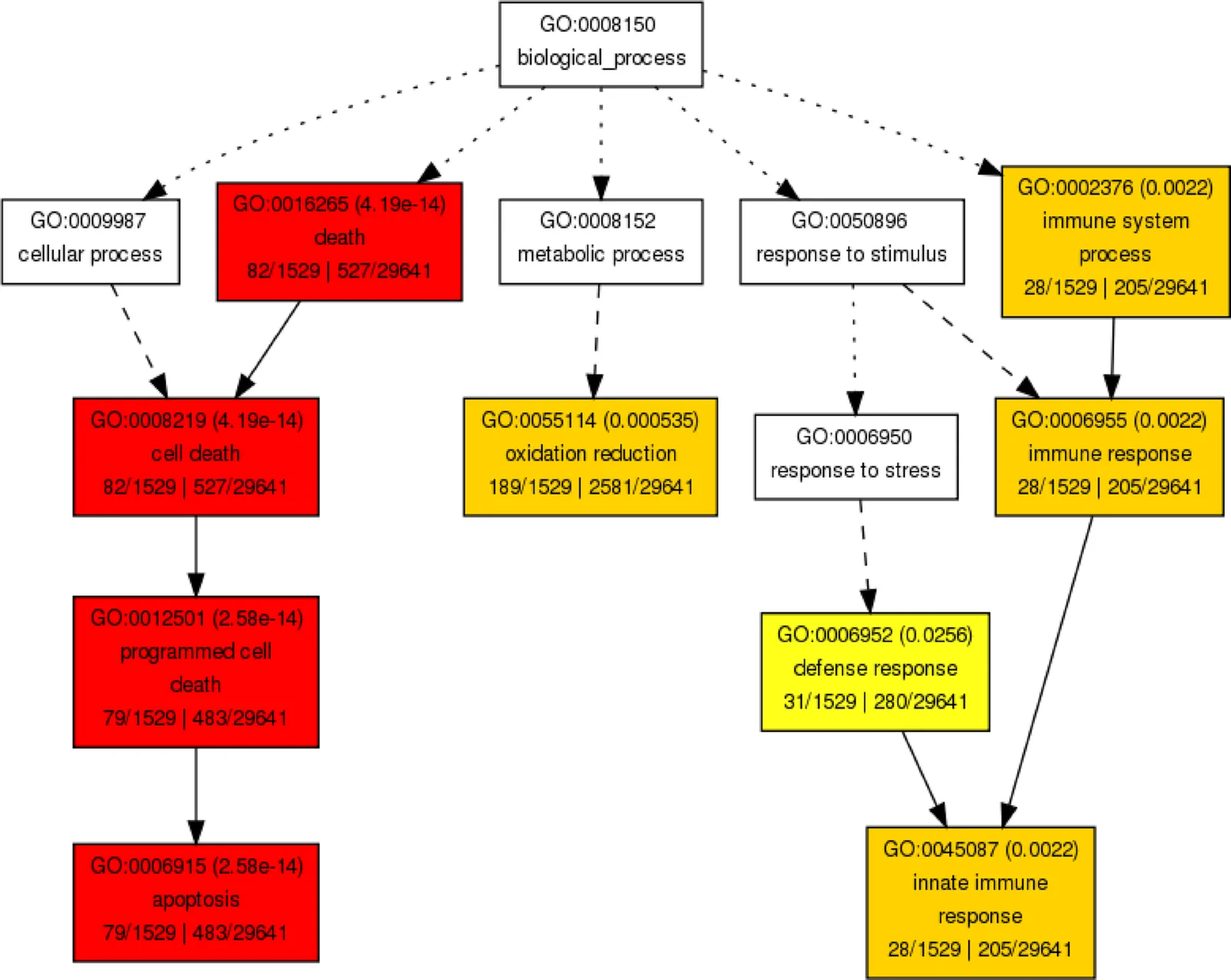
Enriched Gene Ontology analyses of differentially expressed genes (DEGs) between the DAAL (LG13-7552) and the 2n = 40 progeny (LG12-7063) in the biological process category generated from agriGO using DEGs from aligning RNA sequencing reads to the Williams 82 soybean reference genome. Each box contains the GO term number and GO term, if significant, the *p* value in parenthesis, the number of genes in the input list that are associated with the GO term, total number of annotated genes in the input list, the total number of genes in the reference that are associated with the GO term and the total number of genes in the reference are also indicated. The box colors reflect the level of significance of the analysis: yellow < 0.05, orange < 0.005, and red < 0.0005.

GO terms in the biological process category associated with apoptosis, oxidation reduction, and defense and immune response were significant (Fig. 8), which indicates that the 2n = 42 line was functioning under more stress than the 2n = 40 line. This is consistent with the DEG results that showed a large portion of defense genes among the most differentially expressed genes. Whether these stress related genes are causing the phenotypic differences between LG13-7552 and LG12-7063 and the specific trigger to the activation of stress/pathogenic responsive genes and the defense mechanism are yet to be determined. The DAALs were shorter than the 2n = 40 lines and were stunted and distorted in vegetative and reproductive development. If we define stress as any deviation from optimal growth conditions, the enrichment of stress related responses would be expected. The plant tissue used for this RNA-Seq was obtained from young seedlings grown without environmental stress, so the stress experienced by the DAALs would be from genetic rather than environmental factors. The enrichment analysis in the molecular function category of significant receptor activity, nucleoside binding, transporter activity and catalytic activity indicates difficulties in normal cell activity, which also supports our hypothesis that the stress of the extra *G. tomentella* chromosomes suppressed the normal transcription and function of the genes on the 40 soybean chromosomes. This is consistent with observations in mammalian systems that exhibit various stress responses to aneuploidy (Zhu et al. 2018).

It was not the objective of this research to assess the potential of alien addition lines to agronomically improve soybean, but the phenotypic and gene expression data from the DAAL indicates that negative impact of introgression at the chromosomal level. The favorable effects from the introgression of one or a few genes remain to be demonstrated.

### Phenotypic variation among the DAAL-derived progeny population

There are several hypotheses for the chromosomal loss in the DAALs and the associated phenotypic changes among the derived soybean lines. Although DAALs were expected to be stable because the 42 chromosomes are 21 homologous pairs, DAALs failure to transmit the alien chromosomes to progenies had been a primary hypothesis. It is known in various species that not all progenies of DAALs from interspecific hybridizations retain the alien chromosomes (Riley 1960; Islam and Shepherd 1981; Chevre et al. 1991; Littlejohn and Pienaar 1995; Friebe et al. 2000; Kynast et al. 2004; Jauhar et al. 2009; Szakács and Molnár-Láng 2010). If an alien chromosome failed to transmit to the female or male gamete during meiosis, monosomic alien addition lines (2n + 1) or revertants (2n) would be observed among the progenies. The reverted soybean lines should resemble the recurrent parent Dwight, except for *G. tomentella* genes that were introgressed into the soybean genome. However, due to failure to amplify the *I* locus in the *G. tomentella* parent, there was no evidence that this change among the derived soybean lines originated from *G. tomentella*. The variants seen in the *T* and *Dt1* loci are not likely to have originated from *G. tomentella*. Without this evidence, DNA mutation was another possible cause for the phenotypic changes. Wide hybridization to perennial relative *G. tomentella* could have introduced or activated a mutation mechanism. DNA mutations should have occurred at random positions in the genome, and if it mutated a functional gene, a phenotypic change could occur. This hypothesis could explain the phenotypic changes of gray pubescence (*T* to *t*) and determinate growth type (*Dt1* to *dt1*) and was supported by the novel allele at the *T* locus that has not been previously reported. However, genotypic data showed differences between reverted soybean lines and their DAAL progenitors were located on as many as 20 chromosomes, which it is highly unlikely to have occurred by simultaneous mutations.

TEs could cause phenotypic changes. TEs are known for randomly inserting into a functional gene and resulting in phenotypic changes in soybean (Palmer et al. 1989; Bhattacharyya et al. 1997; Zabala and Vodkin 2003, 2008; Nagamatsu et al. 2009) and “jumping” out from a gene could cause differences in gene expression (Xu et al. 2010). Several generations of tissue culture were required to produce these DAAL, which could induce activation of TEs (Peschke et al. 1987; Brettell and Dennis 1991; Peschke and Phillips 1991; Hirochika 1993; Hirochika et al. 1996). This could explain the phenotypic changes from yellow to black seed coat (*I* to *i*). The *I* locus is a multi-CHS gene cluster that induces gene silencing to restrict pigmentation in the seed coat. The failure to amplify fragment #8 in the *I* locus structure verification PCR assay (Clough et al. 2004) could possibly be due to a large insertion in the region that interrupted the pigment restriction. The genotypic data was not sufficient to conclude whether transposons played a key role in the phenotypic changes but like the mutation hypothesis, TEs alone are not likely to cause the high number of genetic changes observed in some lines.

Somaclonal variation that is heritable can result from tissue culture. However, it is now known that culture media that includes 2,4-D that was commonly used in tissue culture media in the past can be a major factor in producing this variation (Garcia et al. 2019). All of the studies previously cited for evidence of TE activation used 2,4-D in their culture medium (Peschke et al. 1987; Brettell and Dennis 1991; Peschke and Phillips 1991; Hirochika 1993; Hirochika et al. 1996) In the cultures that produced the DAALs, plant growth regulators such as 2,4-D were not used so somaclonal variation is unlikely to be a cause for the genetic changes noted in this research.

Another possible cause is cross-pollination in which the reverted soybean lines were F_1_ hybrids between the DAAL and an unknown pollen source. The wide-spread heterozygosity demonstrated by the genotyping of the reverted soybean lines supports this hypothesis. When a DAAL was crossed to a soybean (2n = 40), the F_1_ hybrid has only 41 chromosomes, and the selfed progeny from this MAAL is most likely to lose the extra *G. tomentella* chromosome and be left with 40 soybean chromosomes. In later generations, some of the soybean progenies derived from the DAAL revertants continued to segregate and resulted in the increasing variation in phenotypes among the DAAL-derived population.

If natural cross-pollination caused the changes in phenotypes, the observed frequency of phenotypic change is an estimation of the natural cross-pollination rate among the DAALs. The outcrossing rate in the DAALs varied from 3 to 9% with an over-year average of 4%, which is much higher than documented rates for fertile soybean lines in the literature (Ahrent and Caviness 1994; Beard and Knowles 1971).

Soybean is a self-pollination species, and its natural cross-pollination rate depends on the proximity of the pollen source and presence of pollen vectors. Rates of less than 0.5% between plants in neighboring rows and approximately 1% between plants in close contact have been reported (Weber and Hanson 1961). Some of the phenotypes observed among the revertant lines, such as glabrous plants, curly pubescence, and non-yellow seed coat color are not present in modern soybean cultivars. The most likely pollen source would have been from accessions in the USDA Soybean Germplasm Collection, which were being grown on Crop Science Research and Demonstration Center at the University of Illinois. However, the DAALs that produced the revertant lines were grown between 100 and 500 m from the germplasm plots, depending on the year.

Cross pollination is the most probable cause of the noted variation but the mechanism for allowing for that cross pollination is unknown. Cross pollination rates this high have never been reported in soybean except with the *p*_*2*_ allele that causes incomplete dehiscence of the anthers and cross pollination rates as high as 10% (Bernard and Jaycox 1969; Culbertson and Hymowitz 1990).

A second challenge to this theory is pollen movement in excess of 100 m. Studies in North America demonstrated soybean pollen could move between 10 and 20 m depending on the environment (Boerma and Moradshahi 1975; Nelson and Bernard 1979) but there is no documentation of pollen movement over the distances needed to transfer pollen from germplasm accessions that are the only source of relatively rare alleles that were found in the deviant lines. Further research is needed to determine the genetic stability of DAALs in the absence of pollen vectors and evidence of long-distance pollen movement.

Our observations of deviant lines that seemed to involve changes in only pubescence or stem termination could also be explained by cross pollination. Genotypic data showed multiple polymorphic markers between these lines. In these cases, cross-pollination could have occurred between closely related lines and the resulting progeny had nearly identical phenotypes. However, in these cases cross-pollination is occurring not on DAALs but on 2n = 40 lines derived from the DAALs. All these rates of cross pollination are lower than what we observed with the DAALs but they are still higher than what is typically observed in soybean. This indicated that the genetic change in the DAALs that increased the rate of cross-pollination was still functioning in the revertant plants. Several thousand 2n = 40 lines were derived from the crosses between PI 441001 and Dwight that were not derived from 2n = 42 lines. These lines were genetically stable and none exhibited the genetic changes there were observed in the 2n = 40 lines derived from DAALs. Some of these genetically stable 2n = 40 lines yielded significantly more than Dwight (Akpertey, 2018). The genetic origin of these differences is unknown since, in-depth analysis of some of these lines discovered no evidence of *G. tomentella* DNA within the soybean genome, The observations of increased cross-pollination in 2n = 40 lines derived from the DAALs indicate a genetic change in the soybean genome and that change may have only occurred in 2n = 40 lines that were derived from 2n = 42 lines.

The PCR verification result supported that the seed coat color change within the DAAL-derived 2n = 40 progeny was due to structural changes in the *I* locus, but the inconsistent segregation ratio of seed coat color in the progenies of yellow deviants indicates factors other than the *I* locus were involved. The high percentage of plant rows with poor plant stands indicated that the seeds from the yellow seed coat deviants could have emergence or early survival issues. This might explain the segregation ratio distortion that occurred in several populations. The causal factor is unknown but this is additional evidence that the genome of these 2n = 40 lines has been changed.

In conclusion, the genetic evidence indicates that the changes that were observed in the DAALs, were the result of cross pollination. That conclusion does not provide an explanation for causes of that phenomenon and additional research is needed. What changes occurred in the 2n = 42 lines that allow for much increased rates of cross pollination? What is the mechanism for pollen transfer over distances that have never been documented in soybean or is there another explanation for the rare phenotypes in the derived lines? Are there genetic changes in some of 2n = 40 lines derived from the DAALs that allow for the continued elevated rates for cross-pollination? What is the genetic explanation for the aberrant ratios of black to yellow seed coats in segregating populations derived from 2n = 40 plants? Lines derived from *G. max* by G*. tomentella* crosses remain a fertile field for research.

## Supplementary Information

Below is the link to the electronic supplementary material.

Supplementary Material 1

Supplementary Material 2

Supplementary Material 3

Supplementary Material 4

## Data Availability

The data generated in this study have been submitted to the BioProject PRJNA1404335 titled "Introgression of beneficial traits into soybean by interspecific crossing to Glycine tomentella" in the NCBI Sequence Read Archive (SRA). The accession numbers for the raw reads are SRR36910283, SRR37096522, SRR37096523, SRR37096524, SRR37096525, SRR37096526 and SRR37096527. The Transcriptome Shotgun Assemblies have been deposited at DDBJ/EMBL/GenBank under the accessions GLLV00000000 (*G. tomentella*) and GLLZ00000000 (Derived lines). The versions described in this paper are the first versions, GLLV01000000 and GLLZ01000000, respectively.
