# Supplementary material for: Characterization of disomic alien addition lines from an intersubgeneric cross between Glycine max and G. tomentella and phenotypic variation within those lines and their progenies
Source: Plant Mol Biol. 2026 Aug 28;116(5):91. doi: 10.1007/s11103-026-01727-5 (PMC13525025; doi:10.1007/s11103-026-01727-5)
Supplement: Supplementary file 2 [file 11103_2026_1727_MOESM2_ESM.docx]

Characterization of Disomic Alien Addition Lines from an Intersubgeneric Cross between *Glycine max* and *G. tomentella* and Phenotypic Variation within those Lines and their Progenies

Sufei Wang, Ram Singh, Gopal Battu, Mohammad Belaffif, Steven J. Clough, Matthew Hudson, and Randall Nelson

^1^Department of Crop Sciences, University of Illinois, Urbana-Champaign, IL 61801; ^2^ Current address: Bayer U. S. - Crop Science, Seminis Vegetable Seeds, 500 Lucy Brown Lane, San Juan Bautista, CA 95045. ^3^Current address: HudsonAlpha Institute for Biotechnology, 601 Genome Way Northwest, Huntsville, AL 35806, ^4^Soybean/Maize Germplasm, Pathology, and Genetics Research Unit, US Department of Agriculture–Agricultural Research Service, Urbana, IL 61801; ^5^Soybean/Maize Germplasm, Pathology, and Genetics Research Unit, US Department of Agriculture–Agricultural Research Service, Urbana, IL 61801 (retired). Correspondence may be addressed to Randall Nelson: rlnelson@illinois.edu, Tel: 1-217-333-4346. (https://orcid.org/0000-0001-9482-1763)

**Supplemental Figure S2.** Fluorescent *in situ* hybridization (FISH) and genomic *in situ* hybridization (GISH) of the parental lines, *G.max* cv. Dwight and *G. tomentella* PI 441001. The blue DAPI stained chromosomes are the 1^st^ column. The red FISH images using four Texas red labelled probes to *G. max* centromeric repeats are the 2^nd^ column. The green GISH images using Fluorescein labelled *G. tomentella* genomic DNA are the 3^rd^ column. The merged DAPI/FISH/GISH images are the 4^th^ column. The GISH labels all the *G. tomentella* chromosomes and cross-hybridizes to the Nucleolar Organizer Region (NOR) in the recurrent parent Dwight (white arrow heads). The *G. max* FISH probes label all the 40 chromosomes in Dwight and do not label any of the *G.tomentella* chromosomes. The chromosomes are randomly numbered for counting only and are not genetically designated chromosome numbers. Scale bars are 5 µm. DAPI is 4’,6-diamidino-2-phenylindole.
