# Supplementary material for: Characterization of disomic alien addition lines from an intersubgeneric cross between Glycine max and G. tomentella and phenotypic variation within those lines and their progenies
Source: Plant Mol Biol. 2026 Aug 28;116(5):91. doi: 10.1007/s11103-026-01727-5 (PMC13525025; doi:10.1007/s11103-026-01727-5)
Supplement: Supplementary file 3 [file 11103_2026_1727_MOESM3_ESM.docx]

Characterization of Disomic Alien Addition Lines from an Intersubgeneric Cross between *Glycine max* and *Glycine tomentella*.

Sufei Wang^1,2^, Ram Singh^1^, Gopala Battu^1,3^, Mohammad Belaffif^1,3^, Steven Clough^1,4^ Matthew Hudson^1^, Randall Nelson^1,5^

^1^Department of Crop Sciences, University of Illinois, Urbana-Champaign, IL 61801; ^2^ Current address: Bayer U. S. - Crop Science, Seminis Vegetable Seeds, 500 Lucy Brown Lane, San Juan Bautista, CA 95045. ^3^Current address: HudsonAlpha Institute for Biotechnology, 601 Genome Way Northwest, Huntsville, AL 35806, ^4^Soybean/Maize Germplasm, Pathology, and Genetics Research Unit, US Department of Agriculture–Agricultural Research Service, Urbana, IL 61801; ^5^Soybean/Maize Germplasm, Pathology, and Genetics Research Unit, US Department of Agriculture–Agricultural Research Service, Urbana, IL 61801 (retired). Correspondence may be addressed to Randall Nelson: rlnelson@illinois.edu, Tel: 1-217-333-4346. (https://orcid.org/0000-0001-9482-1763)

| Supplemental Table S1. RepeatMasker summary from blasting all Trinity assembled contigs against eudicots repetitive element database. | | | | | | | | |
| --- | --- | --- | --- | --- | --- | --- | --- | --- |
| sequences: | 133872 |  |  |  | |  |  | |
| total length: | 152424681 | bp | (152424681 bp excl N/X-runs) | | | | | |
| GC level: | 39.88 | % |  |  | |  |  | |
| bases masked: | 10000061 | bp | -6.56% |  | |  |  | |
|  | number of elements^1^ | | length occupied | | | % of sequence | | |
| Retroelements |  | 16105 | 6266656 | | bp | 4.11 | | % |
|  | SINEs: | 232 | 22594 | | bp | 0.01 | | % |
|  | Penelope | 0 | 0 | | bp | 0 | | % |
|  | LINEs: | 4645 | 2021126 | | bp | 1.33 | | % |
|  | CRE/SLACS | 0 | 0 | | bp | 0 | | % |
|  | L2/CR1/Rex | 0 | 0 | | bp | 0 | | % |
|  | R1/LOA/Jockey | 0 | 0 | | bp | 0 | | % |
|  | R2/R4/NeSL | 0 | 0 | | bp | 0 | | % |
|  | RTE/Bov-B | 1472 | 330609 | | bp | 0.22 | | % |
|  | L1/CIN4 | 3176 | 1690705 | | bp | 1.11 | | % |
|  | LTR elements: | 11228 | 4222936 | | bp | 2.77 | | % |
|  | BEL/Pao | 0 | 0 | | bp | 0 | | % |
|  | Ty1/Copia | 6322 | 2189008 | | bp | 1.44 | | % |
|  | Gypsy/DIRS1 | 4615 | 1933928 | | bp | 1.27 | | % |
|  | Retroviral | 0 | 0 | | bp | 0 | | % |
|  |  |  |  | |  |  | |  |
| DNA transposons |  | 6441 | 1326545 | | bp | 0.87 | | % |
|  | hobo-Activator | 1552 | 262180 | | bp | 0.17 | | % |
|  | Tc1-IS630-Pogo | 49 | 4869 | | bp | 0 | | % |
|  | En-Spm | 0 | 0 | | bp | 0 | | % |
|  | MuDR-IS905 | 0 | 0 | | bp | 0 | | % |
|  | PiggyBac | 0 | 0 | | bp | 0 | | % |
|  | Tourist/Harbinger | 582 | 87609 | | bp | 0.06 | | % |
|  | Other (Mirage, P-element, Transib) | 0 | 0 | | bp | 0 | | % |
|  |  |  |  | |  |  | |  |
| Rolling-circles |  | 0 | 0 | | bp | 0 | | % |
| Unclassified: |  | 772 | 268887 | | bp | 0.18 | | % |
|  |  |  |  | |  |  | |  |
| Total Interspersed Repeats: | |  | 7862088 | | bp | 5.16 | | % |
|  |  |  |  | |  |  | |  |
| Small RNA: |  | 260 | 39545 | | bp | 0.03 | | % |
| Satellites: |  | 57 | 4042 | | bp | 0 | | % |
| Simple repeats: |  | 46464 | 1783217 | | bp | 1.17 | | % |
| Low complexity: |  | 6889 | 334417 | | bp | 0.22 | | % |
| ^1^most repeats fragmented by insertions or deletions have been counted as one element | | | | | | | |  |
| The query species was assumed to be eudicotyledons  RepeatMasker version open-4.0.5, sensitive mode run with rm blastn version 2.2.27+  RepBase Update 20140131, RM database version 20140131 | | | | | | | | |
