# Supplementary material for: Characterization of disomic alien addition lines from an intersubgeneric cross between Glycine max and G. tomentella and phenotypic variation within those lines and their progenies
Source: Plant Mol Biol. 2026 Aug 28;116(5):91. doi: 10.1007/s11103-026-01727-5 (PMC13525025; doi:10.1007/s11103-026-01727-5)
Supplement: Supplementary file 4 [file 11103_2026_1727_MOESM4_ESM.docx]

Characterization of Disomic Alien Addition Lines from an Intersubgeneric Cross between *Glycine max* and *Glycine tomentella*.

Sufei Wang^1,2^, Ram Singh^1^, Gopala Battu^1,3^, Mohammad Belaffif^1,3^, Steven Clough^1,4^ Matthew Hudson^1^, Randall Nelson^1,5^

^1^Department of Crop Sciences, University of Illinois, Urbana-Champaign, IL 61801; ^2^ Current address: Bayer U. S. - Crop Science, Seminis Vegetable Seeds, 500 Lucy Brown Lane, San Juan Bautista, CA 95045. ^3^Current address: HudsonAlpha Institute for Biotechnology, 601 Genome Way Northwest, Huntsville, AL 35806, ^4^Soybean/Maize Germplasm, Pathology, and Genetics Research Unit, US Department of Agriculture–Agricultural Research Service, Urbana, IL 61801; ^5^Soybean/Maize Germplasm, Pathology, and Genetics Research Unit, US Department of Agriculture–Agricultural Research Service, Urbana, IL 61801 (retired). Correspondence may be addressed to Randall Nelson: rlnelson@illinois.edu, Tel: 1-217-333-4346. (https://orcid.org/0000-0001-9482-1763)

| Supplemental Table S2. Functional annotations of assembled contigs from Trinity *de novo* assembly using merged RNA sequencing reads of the DAAL (LG12-7063) and the 2n=40 progeny (LG13-7552) that matched *G. tomentella* sequences. All the annotations are from National Center for Biotechnology Information (NCBI) database using blastx. | | | | |
| --- | --- | --- | --- | --- |
| Contig ID^1^ | Annotation from NCBI BLAST^2^ | Description^3^ | % ID^4^ | E-value^5^ |
| c6193_g1_i2 | gi\|113205396\|gb\|ABI34377.1\| | Polyprotein, putative [Solanum demissum] | 41.3 | 1.E-09 |
| c16714_g1_i1 | gi\|147845547\|emb\|CAN78493.1\| | hypothetical protein VITISV_037041 [Vitis vinifera] | 56.6 | 6.E-09 |
| c28016_g1_i1 | gi\|358343207\|ref\|XP_003635698.1\| | hypothetical protein MTR_001s0023 [Medicago truncatula] | 46.9 | 8.E-07 |
| c36149_g2_i1 | gi\|357140780\|ref\|XP_003571941.1\| | PREDICTED: RNA-directed DNA polymerase from mobile element jockey-like [Brachypodium distachyon] | 46 | 4.E-08 |
| c40414_g1_i1 | gi\|357445665\|ref\|XP_003593110.1\| | Zinc finger MYM-type protein [Medicago truncatula] | 62.8 | 2.E-09 |
| c40746_g2_i1 | gi\|147776056\|emb\|CAN69911.1\| | hypothetical protein VITISV_027081 [Vitis vinifera] | 55.9 | 2.E-17 |
| c45590_g2_i1 | gi\|356544228\|ref\|XP_003540556.1\| | PREDICTED: uncharacterized protein LOC100799395 [Glycine max] | 52.3 | 3.E-07 |
| c46986_g1_i1 | gi\|357494985\|ref\|XP_003617781.1\| | hypothetical protein MTR_5g095400 [Medicago truncatula] | 28.5 | 7.E-11 |
| c46986_g1_i2 | gi\|357494991\|ref\|XP_003617784.1\| | hypothetical protein MTR_5g095430 [Medicago truncatula] | 29.2 | 4.E-09 |
| c46986_g1_i4 | gi\|357494985\|ref\|XP_003617781.1\| | hypothetical protein MTR_5g095400 [Medicago truncatula] | 28.5 | 1.E-10 |
| c54260_g1_i2 | gi\|356519637\|ref\|XP_003528477.1\| | PREDICTED: glutamate receptor 2.7-like [Glycine max] | 52.2 | 3.E-18 |
| c55555_g4_i1 | gi\|147772264\|emb\|CAN71870.1\| | hypothetical protein VITISV_044169 [Vitis vinifera] | 75.9 | 2.E-08 |
| c58516_g3_i5 | gi\|87162498\|gb\|ABD28293.1\| | RNA-directed DNA polymerase (Reverse transcriptase); Zinc finger, CCHC-type; Peptidase aspartic, active site; Retrotransposon gag protein [Medicago truncatula] | 80.6 | 1.E-11 |
| c66267_g1_i1 | gi\|357498095\|ref\|XP_003619336.1\| | hypothetical protein MTR_6g046770 [Medicago truncatula] | 35.5 | 9.E-08 |
| c68302_g1_i1 | gi\|396582343\|gb\|AFN88207.1\| | integrase core domain containing protein [Phaseolus vulgaris] | 42.8 | 1.E-24 |
| c71148_g1_i1 | gi\|113205396\|gb\|ABI34377.1\| | Polyprotein, putative [Solanum demissum] | 51.7 | 3.E-10 |
| c73277_g1_i1 | gi\|356514878\|ref\|XP_003526129.1\| | PREDICTED: uncharacterized protein LOC100777620 [Glycine max] | 32.1 | 2.E-08 |
| c76286_g1_i1 | gi\|357503811\|ref\|XP_003622194.1\| | Cellular nucleic acid-binding protein-like protein, partial [Medicago truncatula] | 28 | 1.E-08 |
| c76858_g1_i1 | gi\|356532924\|ref\|XP_003535019.1\| | PREDICTED: uncharacterized protein LOC100795609 [Glycine max] | 33 | 6.E-07 |
| c78066_g1_i1 | gi\|77548423\|gb\|ABA91220.1\| | retrotransposon protein, putative, unclassified [Oryza sativa Japonica Group] | 64.9 | 5.E-08 |
| ^1^ Identifier of the assembled contigs from Trinity *de novo* assembly;  ^2^ Functional annotations of the assembled contigs from NCBI database;  ^3^ Descriptions of the functional annotations of the assemblyed contigs;  ^4^ The probability of the functional annotations and the assembled contig sequences were matched by chance;  ^5^ Percent identity between the functional annotations and the assembled contigs. | | | | |
